# Care after pancreatic resection according to an algorithm for early detection and minimally invasive management of pancreatic fistula versus current practice (PORSCH-trial): design and rationale of a nationwide stepped-wedge cluster-randomized trial

**DOI:** 10.1186/s13063-020-4167-9

**Published:** 2020-05-07

**Authors:** F. Jasmijn Smits, Anne Claire Henry, Casper H. van Eijck, Marc G. Besselink, Olivier R. Busch, Mark Arntz, Thomas L. Bollen, Otto M. van Delden, Daniel van den Heuvel, Christiaan van der Leij, Krijn P. van Lienden, Adriaan Moelker, Bert A. Bonsing, Inne H. M. Borel Rinkes, Koop Bosscha, R. M. van Dam, Sebastiaan Festen, B. Groot Koerkamp, Erwin van der Harst, Ignace H. de Hingh, Geert Kazemier, Mike Liem, B. Marion van der Kolk, Vincent E. de Meijer, Gijs A. Patijn, Daphne Roos, Jennifer M. Schreinemakers, Fennie Wit, C. Henri van Werkhoven, I. Quintus Molenaar, Hjalmar C. van Santvoort, for the Dutch Pancreatic Cancer Group

**Affiliations:** 1 Department of Surgery, Regional Academic Cancer Center Utrecht, St. Antonius Hospital and University Medical Center Utrecht, PO Box 85500, Utrecht, 3508 GA The Netherlands; 2 Department of Surgery, Erasmus Medical Center, Rotterdam, The Netherlands; 3 Department of Surgery, Cancer Center Amsterdam, Amsterdam UMC, University of Amsterdam, Amsterdam, The Netherlands; 4 Department of Radiology, Radboud University Medical Center, Nijmegen, The Netherlands; 5 Department of Radiology, St. Antoniusziekenhuis, Nieuwegein, The Netherlands; 6 Department of Radiology, Cancer Center Amsterdam, Amsterdam UMC, University of Amsterdam, Amsterdam, The Netherlands; 7 Department of Radiology, Maastricht University Medical Center, Maastricht, The Netherlands; 8 Department of Radiology, Erasmus Medical Center, Rotterdam, The Netherlands; 9 Department of Surgery, Leiden University Medical Center, Leiden, The Netherlands; 10 Department of Surgery, Jeroen Bosch Ziekenhuis, ´s-Hertogenbosch, The Netherlands; 11 Department of Surgery, Maastricht University Medical Center, Maastricht, The Netherlands; 12 Department of Surgery, Onze Lieve Vrouwe Gasthuis, Amsterdam, The Netherlands; 13 Department of Surgery, Maasstad Ziekenhuis, Rotterdam, The Netherlands; 14 Department of Surgery, Catharina Ziekenhuis, Eindhoven, The Netherlands; 15 Department of Surgery, Cancer Center Amsterdam, Amsterdam UMC, VU University, Amsterdam, The Netherlands; 16 Department of Surgery, Medisch Spectrum Twente, Enschede, The Netherlands; 17 Department of Surgery, Radboud University Medical Center, Nijmegen, The Netherlands; 18 Department of Surgery, University of Groningen and University Medical Center Groningen, Groningen, The Netherlands; 19 Department of Surgery, Isala Ziekenhuis, Zwolle, The Netherlands; 20 Department of Surgery, Reinier de Graaf Gasthuis, Delft, The Netherlands; 21 Department of Surgery, Amphia Ziekenhuis, Breda, The Netherlands; 22 Department of Surgery, Tjongerschans, Heerenveen, The Netherlands; 23 Julius Center for Health Sciences and Primary Care, University Medical Center Utrecht, Utrecht University, Utrecht, The Netherlands

## Abstract

**Background:**

Pancreatic resection is a major abdominal operation with 50% risk of postoperative complications. A common complication is pancreatic fistula, which may have severe clinical consequences such as postoperative bleeding, organ failure and death. The objective of this study is to investigate whether implementation of an algorithm for early detection and minimally invasive management of pancreatic fistula may improve outcomes after pancreatic resection.

**Methods:**

This is a nationwide stepped-wedge, cluster-randomized, superiority trial, designed in adherence to the Consolidated Standards of Reporting Trials (CONSORT) guidelines. During a period of 22 months, all Dutch centers performing pancreatic surgery will cross over in a randomized order from current practice to best practice according to the algorithm. This evidence-based and consensus-based algorithm will provide daily multilevel advice on the management of patients after pancreatic resection (i.e. indication for abdominal imaging, antibiotic treatment, percutaneous drainage and removal of abdominal drains). The algorithm is designed to aid early detection and minimally invasive step-up management of postoperative pancreatic fistula. Outcomes of current practice will be compared with outcomes after implementation of the algorithm. The primary outcome is a composite of major complications (i.e. post-pancreatectomy bleeding, new-onset organ failure and death) and will be measured in a sample size of at least 1600 patients undergoing pancreatic resection. Secondary endpoints include the individual components of the primary endpoint and other clinical outcomes, healthcare resource utilization and costs analysis. Follow up will be up to 90 days after pancreatic resection.

**Discussion:**

It is hypothesized that a structured nationwide implementation of a dedicated algorithm for early detection and minimally invasive step-up management of postoperative pancreatic fistula will reduce the risk of major complications and death after pancreatic resection, as compared to current practice.

**Trial registration:**

Netherlands Trial Register: NL 6671. Registered on 16 December 2017.

## Background

Without treatment, survival in patients with pancreatic cancer is only 4–6 months. Pancreatic resection combined with adjuvant chemotherapy can increase the median survival to 54 months in selected patients with pancreatic cancer [1]. Pancreatic resection is, however, a major abdominal operation that is associated with a 50% risk of complications [2]. Complications are associated with postoperative death and reduced long-term survival because patients with a complicated postoperative course are often unfit to receive adjuvant chemotherapy [3–5].

A common complication after pancreatic resection is pancreatic fistula, in which there is leakage of pancreatic juices into the abdominal cavity [6–9]. This complication is potentially life threatening due to its association with other complications. Leakage of pancreatic juices can lead to vessel erosion, potentially causing major bleeding. Systemic inflammation may result in sepsis, organ failure and death [8]. A postoperative pancreatic fistula that necessitates a change in postoperative management is regarded a “clinically relevant fistula” [10]. In a recent systematic review of 40 studies, clinically relevant pancreatic fistula occurred in 13% of patients after pancreatic resection [2]. A Dutch study (2005–2013) demonstrated that mortality in patients with severe pancreatic fistula remains as high as 18% [9]*.*

In general, in-hospital mortality after pancreatoduodenectomy varies widely among Dutch centers (interquartile range 0–8%). This variability appears to be explained by differences in failure-to-rescue rates between centers (i.e. mortality in patients with major postoperative complications) [11]. A recent multicenter propensity-score-matched study by the Dutch Pancreatic Cancer Group identified doubling of mortality after primary relaparotomy, as compared with primary minimally invasive catheter drainage for postoperative pancreatic fistula (36% vs. 14%, *P* = 0.007; risk ratio 0.39; 95% confidence interval 0.20–0.75) [9]. Relaparotomy causes a major inflammatory response syndrome in patients who are already ill, which might induce or aggravate multiple organ failure and possibly death [12]. As an alternative to relaparotomy, minimally invasive percutaneous catheter drainage has been shown to be very effective in the majority of patients [9]. In daily practice, however, both treatment strategies are still frequently performed (primary catheter drainage 73%, primary relaparotomy 27%) with no change towards a more minimally invasive management strategy [9]. This might be related to the lack of (international) guidelines on postoperative patient monitoring and management of complications.

This trial is designed to investigate whether implementation of an algorithm for postoperative care focusing on early detection and minimally invasive step-up management of postoperative pancreatic fistula results in a lower rate of major complications and death after pancreatic resection, as compared with current practice. This trial will also evaluate the superiority of the algorithm in other clinical outcomes, the number of patients receiving chemotherapy, quality of life, healthcare resource utilization and costs.

## Methods

### Trial design

**Fig. 1:**
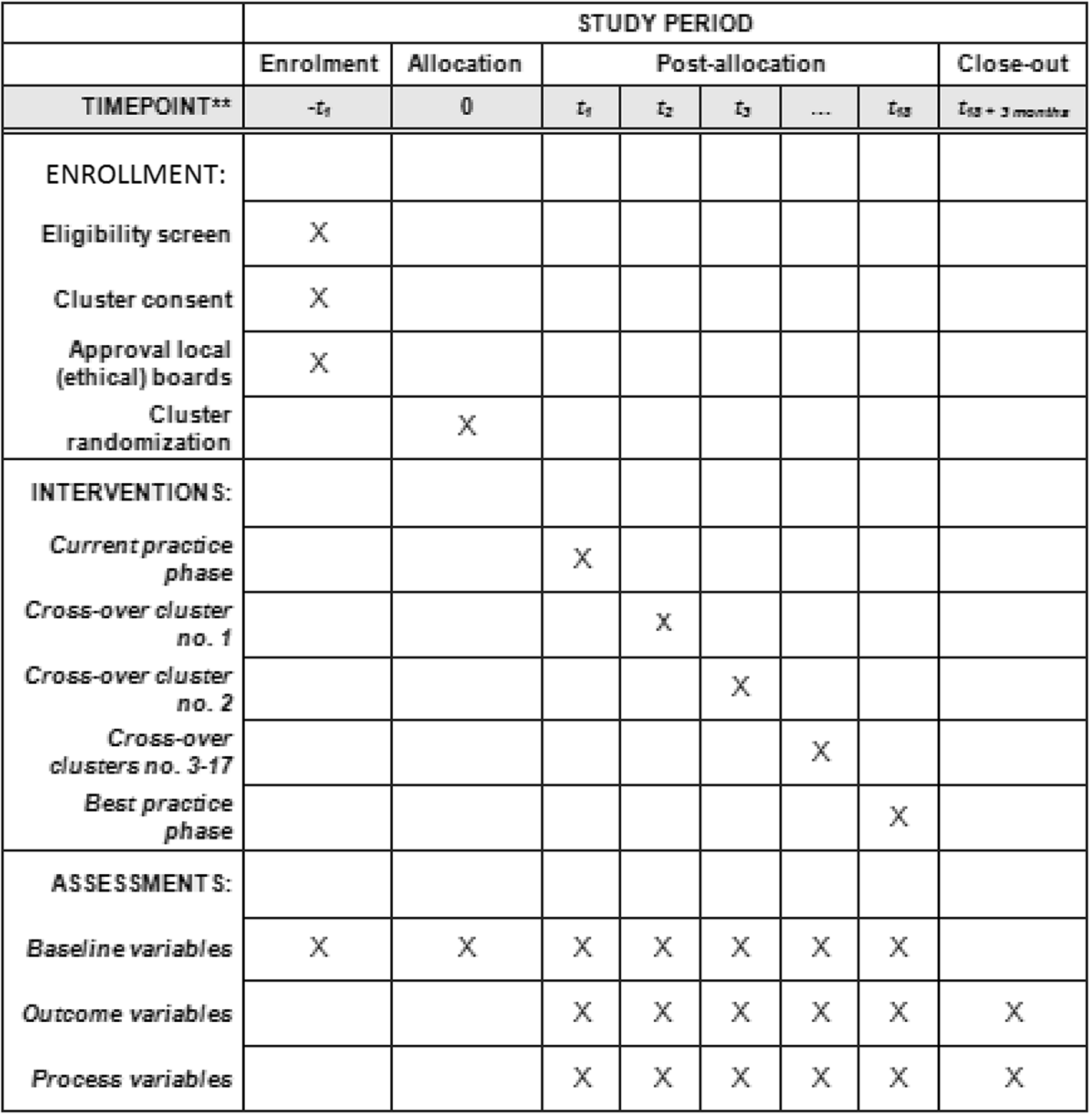
Standard Protocol Items: Recommendation for Interventional Trials (SPIRIT) schedule of enrollment, interventions and assessments

This is a nationwide, stepped-wedge, cluster-randomized trial in all centers of the Dutch Pancreatic Cancer Group. The research team will implement the algorithm for early detection and minimally invasive management of pancreatic fistula to change professional behavior in each hospital and therefore stepped-wedge cluster randomization at center level will be performed. This trial was designed in accordance with the Consolidated Standards of Reporting Trials (CONSORT) guidelines for stepped-wedge, cluster-randomized trials [13] and the Standard Protocol Items: Recommendation for Interventional Trials (SPIRIT) guidelines for clinical trials (Fig. 1) [14].

**Fig. 2:**
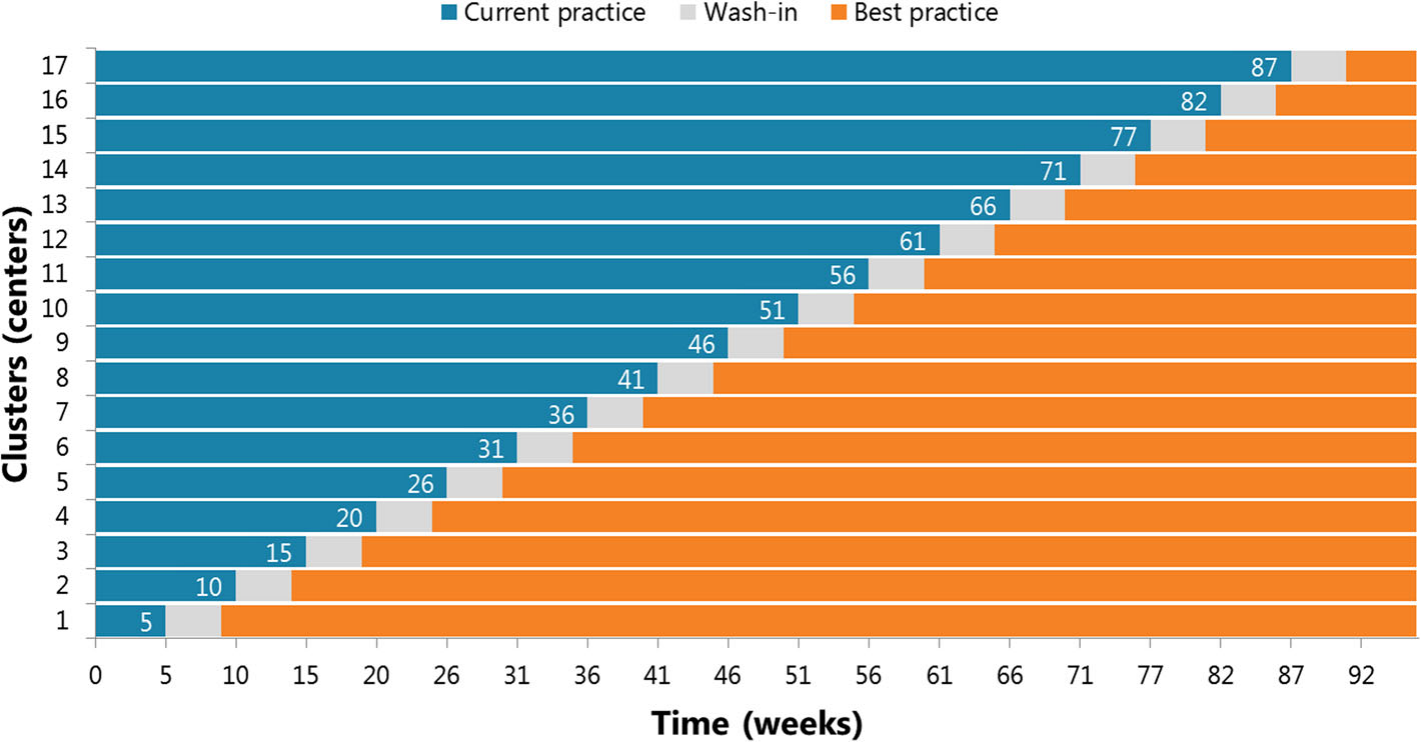
Schematic overview of trial design

The stepped-wedge, cluster-randomized trial is a form of cross-over design with unidirectional cross-over (from control to intervention phase) at different time points for each cluster (Fig. 2). Each cluster will contain one center. Randomization will determine the order in which each center will undergo the transition [15, 16]. In the first timeframe, all centers will deliver current practice (i.e. control). In the second timeframe, the center in the first cluster will start with algorithm-based best practice (i.e. intervention), while all other centers will still deliver current practice. In the third timeframe, the second cluster will implement the algorithm. Following this approach, we proceed until all centers have stepped over to the algorithm. In the last timeframe, all centers will deliver treatment according to the algorithm. Each cluster will contain one center; therefore, the number of sequences is equal to the number of participating centers and this will be 17. To ensure adequate implementation of the algorithm, a wash-in period was designed in which clinicians in the center will be intensively trained by the research team on the algorithm. To avoid contamination of centers still in the current-practice phase, local clinicians will only have access to the algorithm after cross-over to care according to the algorithm.

### Participants

Pancreatic surgery is centralized in The Netherlands in centers performing at least 20 pancreatoduodenectomies annually. All centers performing pancreatic surgery in The Netherlands are participating in this trial. All patients undergoing all types of pancreatic resection in these centers are identified and will be recruited in this trial. There are no exclusion criteria for centers or patients (i.e. nationwide complete enumeration).

Data will be extracted from the prospective Dutch Pancreatic Cancer Audit. Additional data on clinical outcomes will be extracted prospectively through continuous systematic evaluation of patient charts using a predefined case record form. Data are stored on a central secured data capturing tool (i.e. Castor EDC) containing programmed checks for outliers. To ensure patient confidentiality, the data stored centrally will be cleaned to remove all information that could directly trace back to an individual patient, and a unique patient identifier number will be assigned to every patient. The local principal investigator will have access to the identifiable patient information. The data entered will be checked by a senior researcher. Local principal investigators are responsible for data collection. They can delegate this to the study personnel if resources are not available at the center.

### Withdrawal and replacement of centers

For the design of this trial it is essential that all participating centers participate for the entire duration of the study, to prevent an unequal distribution of patients across the two study arms (i.e. before and after implementation). If a center stops performing pancreatic surgery during the study the randomization order will be maintained. All patients treated in this center will still be included in the final analysis, according to intention-to-treat analysis. As all centers performing pancreatic surgery in the Netherlands are participating in this trial, centers will not be replaced after withdrawal.

### Intervention

This trial evaluates a nationwide cluster-level intervention in which all clinicians involved in the postoperative care of patients undergoing pancreatic resection are educated on the new algorithm for early detection and minimally invasive management of postoperative pancreatic fistula. Currently, guidelines on how to diagnose and manage postoperative pancreatic fistula are lacking. This is concerning, since management of complications appears to be the most important factor in decreasing mortality after pancreatic resection. The algorithm is based on findings in Dutch observational cohort studies, comprehensive systematic literature analyses, an inventory of current guidelines on postoperative care and expert opinion. The impact of the proposed algorithm was evaluated in a retrospective multicenter cohort and consensus upon this algorithm was reached in several plenary meetings with one pancreatic surgeon from each of the centers of the Dutch Pancreatic Cancer Group. The final algorithm was reviewed by the advisory committee of respected international experts from centers performing a high volume of pancreatic resections before implementation in this trial. As a result, we have created a best-practice algorithm based on national and international consensus. A complete overview of the design and details on the content of the algorithm are provided in Additional file 1: Appendix 1.

**Fig. 3:**
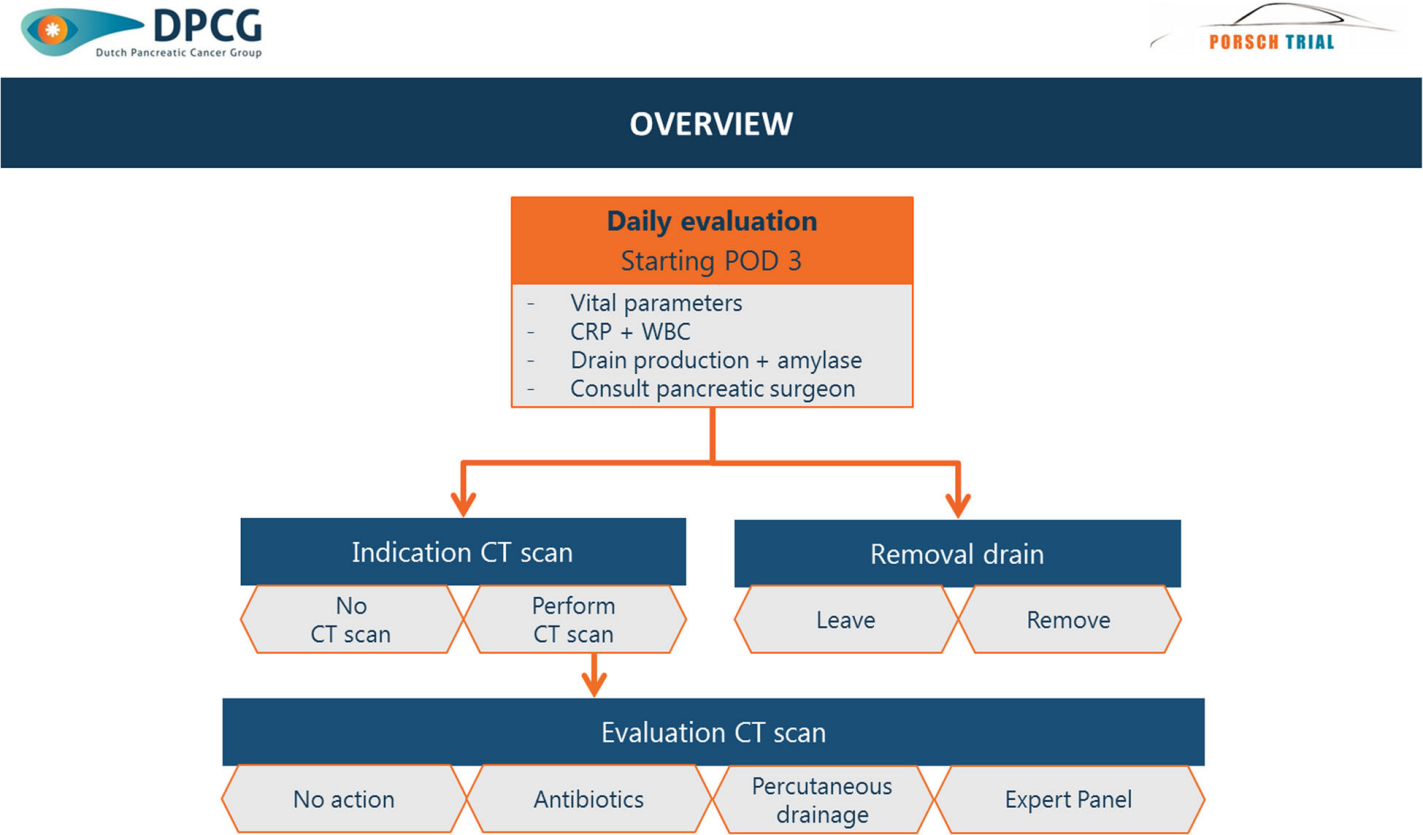
Schematic overview of algorithm. Schematic overview of the study algorithm. Daily evaluation starts on postoperative day (POD) 3 and includes vital parameters (i.e. temperature, heart rate, respiratory rate), C-reactive protein (CRP), white blood cell count (WBC), drain production, drain amylase level and a daily consult by a pancreatic surgeon. The algorithm will provide advice on three levels: indication of abdominal computed tomography (CT) scan, removal of abdominal drain(s), indication for (invasive) intervention based on systematic evaluation of abdominal CT scan

**Fig. 4:**
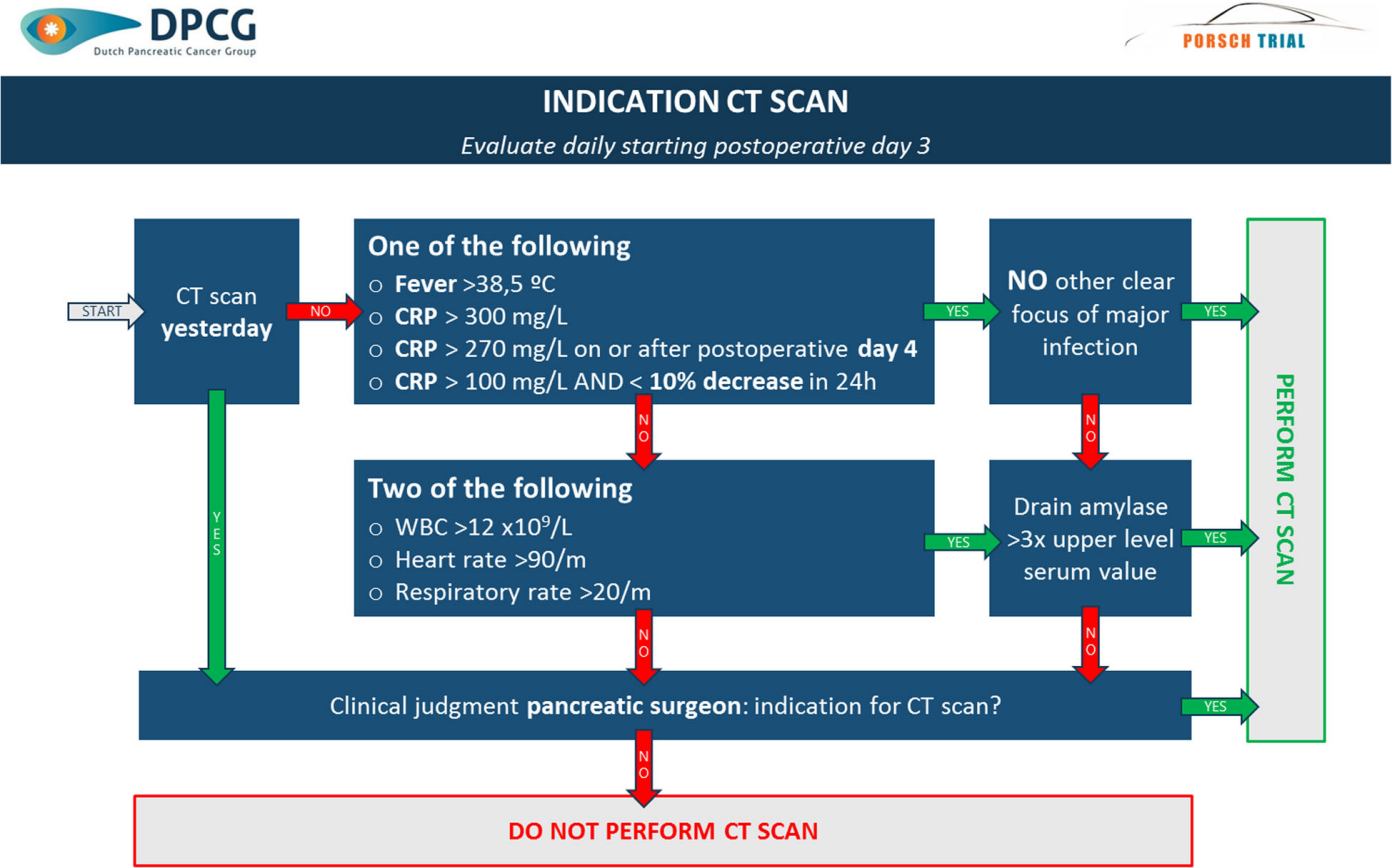
Indication for computed tomography (CT) scan. Schematic overview of the indication for abdominal CT scan. °C, degrees Celsius; CRP, C-reactive protein; mg, milligram; L, liter; H, hours; WBC, white blood cell count; M, minute

**Fig. 5:**
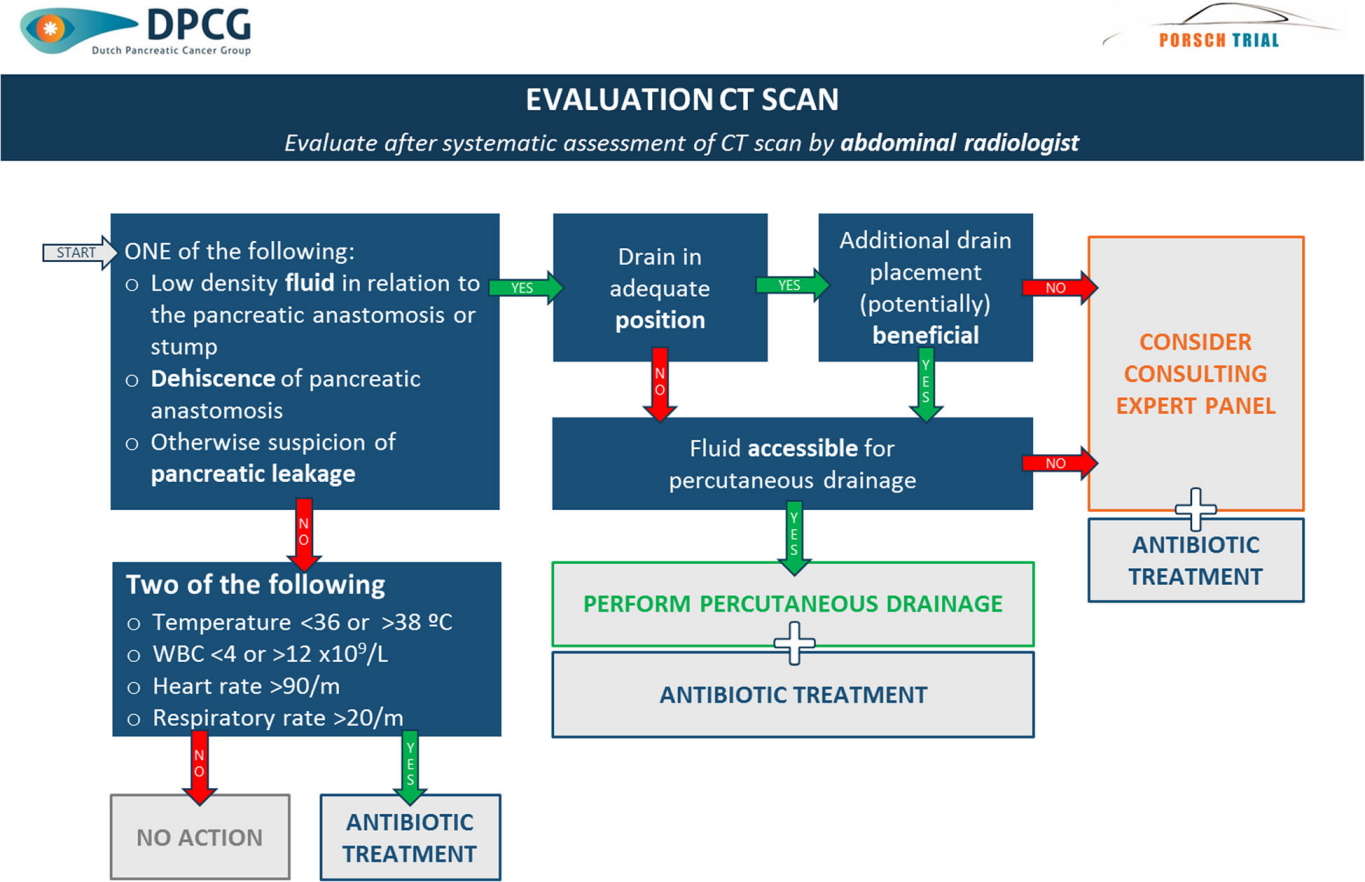
Evaluation of computed tomography (CT) scan. Schematic overview of the indication for (invasive) intervention based on systematic evaluation of abdominal CT scan. °C, degrees Celsius; CRP, C-reactive protein; L, liter; h, hours; WBC, white blood cell count; m, minute

**Fig. 6:**
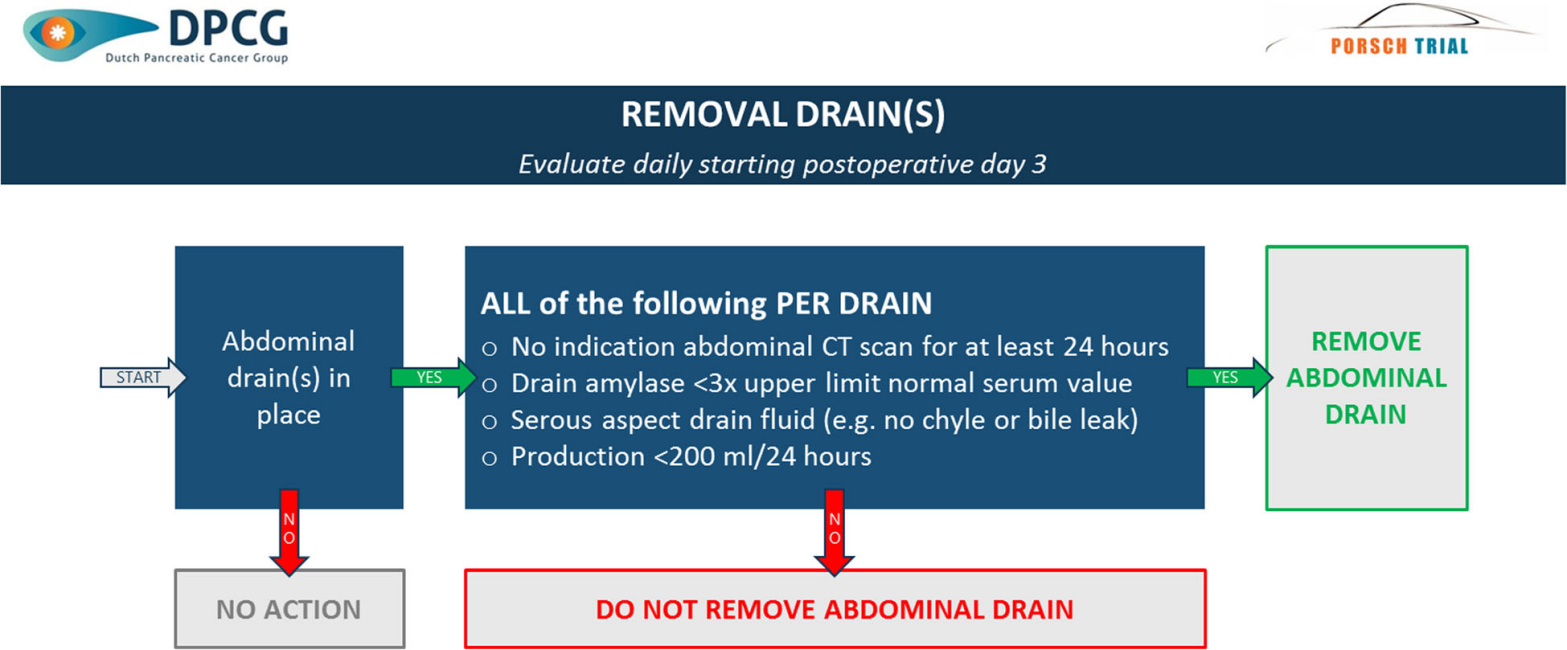
Removal of abdominal drains. Schematic overview of the removal of abdominal drain(s). ml, milliliter

A schematic overview of the multilevel algorithm is provided in Fig. 3. It is based on daily standardized evaluation of all patients undergoing pancreatic resection from postoperative day 3 to discharge up to a maximum of 14 days. The algorithm will provide daily advice on three decision levels: indication for abdominal computed tomography (CT) scan (Fig. 4); indication for (invasive) intervention (i.e. minimally invasive percutaneous drainage and/or antibiotic treatment) based on evaluation of the abdominal CT scan (Fig. 5) and indication for removal of abdominal drain(s) (Fig. 6). The algorithm includes clinical data from physical examination and biochemical tests (i.e. systemic inflammatory response syndrome (SIRS), C-Reactive protein (CRP), white blood cell count (WBC), drain production including amylase content and a daily consult from a pancreatic surgeon). At predefined, evidence-based cutoff points (Fig. 4), an abdominal contrast-enhanced CT scan and possibly subsequent minimally invasive intervention should be performed or antibiotic treatment should be started (Fig. 5). Each CT scan will be evaluated according to predefined criteria (Fig. 5) to assess whether there is suspicion of a pancreatic fistula and whether there is an indication for minimally invasive percutaneous catheter drainage. In the case of no clinical improvement within 48 h after the last CT scan, a further CT scan should be performed to assess the indication for and possibility of (additional invasive) intervention. The indication to remove abdominal drains will be evaluated daily starting on postoperative day 3, for as long as there are abdominal drains in place (Fig. 6). The interpretation of the algorithm, the rationale behind the different proposed cutoff points and the proposed management of bleeding or hepaticojejunostomy leakage is provided in Additional file 1: Appendix 2. Since we propose an intervention of structured use of diagnostic and invasive interventions that are already used in daily practice, no additional harms to patients are introduced in this study.

To facilitate clinical use of this complex algorithm, it was incorporated in a smartphone application that should be filled out daily for every patient. Access to this application was granted to all clinicians participating in the trial. The application will provide multilevel advice based on the clinical and biochemical parameters entered daily. The application will not contain information that can be traced back to an individual patient. All data will be stored on a secure central database that is accessible to the study coordinators 24 h a day, 7 days a week. This was used to evaluate adherence to the study protocol. Local caregivers are contacted and reminded of the application when it is not filled out for every patient in the early afternoon, so that CT and potential drainage (if indicated) can still be performed on the same day.

Since clinical decision-making on invasive intervention in these patients is challenging, an expert panel is available to assess the indication for and feasibility of percutaneous drainage in the management of pancreatic fistula. This online panel, consisting of dedicated radiologists with expertise in abdominal radiology, interventional radiologists and surgeons with expertise in pancreatic surgery, is available 24 h a day, 7 days a week. The advice will be reported back to the clinician within 12 h after consultation or when at least three experts provide their advice on the next step in the management of these patients. The organization of this expert panel will be similar to the Dutch Pancreatitis Study Group expert panel [17].

### Outcomes

All outcomes are assessed up to 90 days after the index pancreatic resection or, if admission exceeds 90 days, up to hospital discharge. All relevant definitions are provided in Table 1. The primary endpoint is a composite of the following three major complications occurring after study intervention:

- New-onset postoperative bleeding requiring invasive intervention
- New-onset organ failure (i.e. pulmonary, circulatory or renal)
- Death

**Table 1:** Relevant definitions

New onset	Not present any time in 24 h before study intervention; accounts for all outcomes
Mortality	Rate of death occurring within 90 days after index pancreatic resection or, if index admission exceeds 90 days, during admission
Organ failure [12]	
Pulmonary	PaO2 < 60 mmHg, despite FiO2 of 0.3, or need for mechanical ventilation
Circulatory	Systolic blood pressure < 90 mmHg, despite adequate fluid resuscitation, or need for inotropic support
Renal	Creatinine level > 177 μmol/liter after rehydration or need for hemofiltration or hemodialysis
Post-pancreatectomy hemorrhage (PPH)	Adapted from Wente et al [18]
Grade A	Occurring < 24 h after pancreatectomy (*early*) with no therapeutic consequences
Grade B	Both early (< 24 h) and late (> 24 h) requiring therapy (including fluid or blood transfusion and transfer to high-care unit), with non-life-threatening clinical condition. Includes early PPH requiring relaparotomy
Grade C	Occurring > 24 h after pancreatectomy (*late*) with severely impaired, life-threatening clinical condition requiring intervention
Comprehensive Complication Index (CCI)	This summarizes all postoperative complications, other than pre-existing complications, in a score from 0 (no complications) to 100 (death). The CCI can be readily computed on the basis of tabulated complications according to the Clavien-Dindo classification [19, 20]
Postoperative pancreatic fistula	Amylase in drain fluid on or after postoperative day 3 of at least three times the upper level of normal serum amylase [21]
Biochemical leak	Requiring no change in postoperative management, hospital stay not prolonged
Grade B	Persistent drainage > 3 weeks, change in postoperative management (i.e. catheter drainage, or angiographic procedure for bleeding, signs of infection without organ failure, no relaparotomy), all related to pancreatic fistula
Grade C	Grade B with reoperation, organ failure or death related to pancreatic fistula
Postoperative bile leakage	Bilirubin in drain fluid on or after postoperative day 3 of at least three times the upper level of normal serum bilirubin (adapted from Koch [22])
Delayed gastric emptying	Adapted from Wente et al [23]
Grade A	Nasogastric tube postoperative day 4–7 or need for replacement of tube after postoperative day 3; oral intake between day 7 and 14
Grade B	Nasogastric tube postoperative day 8–14 or need for replacement of tube after postoperative day 7; oral intake between day 14 and 21
Grade C	Nasogastric tube after postoperative day 14 or need for replacement of tube after postoperative day 14; oral intake after day 14
Gastroenterostomy leakage	As seen on abdominal imaging or during relaparotomy or secretion of fecal material from percutaneous drain or through surgical wound
Acute pancreatitis	Combination of abdominal pain, threefold increased amylase and lipase levels or as seen on radiologic imaging [24]
New-onset diabetes mellitus	Need for insulin or oral diabetes drugs within 3 months after discharge, not present before pancreatoduodenectomy
Exocrine pancreatic insufficiency	Need for oral pancreatic-enzyme supplementation within 3 months after discharge, not present before pancreatoduodenectomy
Body mass index	Weight in kilograms divided by the square of the height in meters
ASA	American Society of Anesthesiologists classification
I	Healthy patient without systemic disease
II	Patient with mild systemic disease
III	Patient with severe systemic disease, limiting activity but not life-threatening

*PaO2* arterial partial pressure of oxygen, *FiO2* fraction of inspired oxygen

The rationale for this primary endpoint is that the proposed algorithm for early detection and step-up management of pancreatic fistula can possibly prevent clinical deterioration. The most clinically severe complications associated with pancreatic fistula are combined in this endpoint [8]. Members of an adjudication committee will individually asses the primary endpoint while blinded to the assigned treatment arm; disagreements will be resolved by consensus discussions, with allocation concealment.

Secondary endpoints include individual components of the composite endpoint and an adapted version of the primary endpoint in which only complications are included that are deemed by the blinded adjudication committee to be directly related to a pancreatic fistula. Other secondary endpoints are: the Comprehensive Complication Index (CCI) [25] based on complications grade 3 or higher according to the Clavien-Dindo classification [19], postoperative pancreatic fistula [21], gastroenterostomy leakage, postoperative bile leakage [22], delayed gastric emptying [23], chyle leakage [26], new-onset acute pancreatitis [24], number and timing of abdominal CT scans, number, timing and type of invasive (re-)interventions, admission to the Intensive Care Unit (ICU), length of ICU stay, length of hospital stay, readmission rate, number of patients receiving adjuvant chemotherapy at 90 day follow up, duration of postoperative pancreatic fistula (time to removal of the last abdominal drain or completion of pancreatectomy), success of implementation (i.e. based on number and timing of abdominal CT scans and proportion of patients’ days in which the algorithm was not followed). Daily data on the incidence and duration of SIRS, sepsis and organ failure (according to the definitions in Table 1 and according to Sequential Organ Failure Assessment (SOFA) and the Multiple Organ Dysfunction Score (MODS)) are also collected [27–29]. In addition, we aim to evaluate the impact on quality of life, total direct and indirect costs and budget impact (see “Economic evaluation”).

### Sample size calculation

The PORSCH trial is a superiority trial. The effect of the intervention will be measured using the incidence of the primary endpoint (i.e. post-pancreatectomy bleeding, new-onset organ failure or death), which is expected to become lower after implementation of the algorithm. In this study, we aim to improve the outcomes of all patients undergoing pancreatectomy. However, to determine the superiority of the algorithm in a homogenous sample of patients, the sample size calculation was based on the cohort of patients undergoing pancreatoduodenectomy.

For sample size calculation we evaluated three Dutch datasets: the mandatory Dutch Pancreatic Cancer Audit (*n* = 1686), data from the study on management of pancreatic fistula (*n* = 309 patients [9]) and the validation study performed in preparation for this trial (*n* = 174). All outcome data used for sample size calculation are presented in Table 2. The composite primary endpoint occurred in 13.8% of patients in the Dutch cancer audit (2014–2015), in 44% of patients with severe pancreatic fistula after pancreatoduodenectomy [9] and in 17% in our own validation database (2016). For sample size calculation we used the lowest incidence that we identified in these three datasets (i.e. 13.8% from the audit data).

**Table 2:** Composite primary endpoint

	DPCA	Management of pancreatic fistula	Validation database
Year(s)	2014–2015	2005–2013	2016
Patients	1686	309	174
Composite primary endpoint	12%	44%	15%
In patients undergoing pancreatoduodenectomy	14%	44%	17%
In patients undergoing distal pancreatectomy	7%	–	7%
Relative reduction between study groups	–	53%	–
Relative reduction between quartiles	62%	–	–

Incidence of the primary composite endpoint in three databases; “years” represents the time pancreatic resections were performed; “patients” represents the number of patients included in the analysis. “Management of pancreatic fistula” refers to Smits et al. JAMA Surg [9]. and includes only patients with severe pancreatic fistula; “validation study” refers to data used to validate the proposed algorithm

*DPCA* Dutch Pancreatic Cancer Audit

The expected reduction in the composite primary endpoint was evaluated in two datasets. A relative reduction of 53% was observed in the study on management of pancreatic fistula (i.e. 34% after primary catheter drainage versus 73% after primary relaparotomy). Using the Dutch Pancreatic Cancer Audit data, centers were divided into four quartiles based on the incidence of the primary endpoint after pancreatoduodenectomy. The incidence was 21% in the worst-scoring quartile containing 292 patients from four centers. The incidence of the composite primary endpoint was 8% (i.e. relative reduction 62%) in the best-scoring quartile containing 331 patients who underwent pancreatoduodenectomy in five centers. Based on these outcomes, a relative reduction of 50% in the primary endpoint was used for the sample size in the PORSCH trial. This was the minimal reduction deemed clinically relevant by the members of the trial steering committee and stakeholders from the Dutch Pancreatic Cancer Group.

The required sample size was calculated using the formula for stepped-wedge designs using an expected incidence of 13.8%, a relative reduction of 50%, two-sided alpha of 0.05 and power of 0.80 [30]. The intra-cluster correlation (ICC) estimated from the Dutch Pancreatic Cancer Audit was 0.009 (95% CI 0.006–0.049). As the within-period and between-period ICC were the same, the cluster autocorrelation was set at 1 [31]. Table 3 provides the required sample size for different numbers of participating sites (i.e. clusters). This number dictates the inclusion time in this study design, which was based on the total number of pancreatoduodenectomies performed in the last 2 years according to the Dutch Pancreatic Cancer Audit. As each cluster will contain one of the 17 participating centers, the number of sequences is equal to the number of clusters. Based on the annual number of pancreatoduodenectomies performed in The Netherlands in 2014 and 2015, the planned study duration is 92 weeks to include the required sample size of 1186 pancreatoduodenectomies with complete enumeration (i.e. 5.1 weeks per step for 18 time periods). The total duration of the trial will be 96 weeks, including a 4-week wash-in period.

**Table 3:** Required sample size for different numbers of participating centers

Number of centers	Cluster size^**a**^	Required sample size	Expected inclusions	Inclusion time^**b**^
14	5.9	1239	1239	28
15	5.1	1220	1224	25
16	4.5	1204	1224	24
17	3.9	1186	1193	22

^a^ Average number of patients per center for every step in the stepped-wedge design

^b^ Inclusion time in months including 4 weeks wash-in phase

As we include patients undergoing all types of pancreatic resection in this trial, the total number of patients registered in this trial is expected to be 25% more than the required sample size. We will perform the primary analyses in patients undergoing pancreatoduodenectomy and subsequently in all patients undergoing any type of pancreatic resection.

### Interim analysis

An interim analysis will be performed to evaluate the inclusion rate. At 50% of the planned time for inclusion, the total number of inclusions will be evaluated. If the sample size is less than 47.5% of the target at that time, the duration of steps in the design for the remaining part of the study will be prolonged such that power of 80% is maintained. If the sample size is adjusted at interim analysis, the adjusted dates for cross-over will dictate the allocation of patients to routine practice or best practice for the analysis.

### Safety reporting

No experimental interventions are introduced in this trial. Therefore, no additional safety or health risks are introduced for patients within this trial as compared to regular care and therefore no specific safety monitoring is performed.

### Randomization, blinding and treatment allocation

Randomization is performed after obtaining consent for participation in this trial from the principal investigator and after approval from local (medical ethical) committees of every participating center was obtained. Centers are randomized by an independent statistician using R statistics software to determine the timing of cross-over from current practice to the algorithm [32]. Stratification at randomization is applied for center volume (>45 vs. ≤ 45 pancreatic resections a year, median value based on data from the Dutch Pancreatic Cancer Audit 2014–2015).

Local principal investigators are informed about the allocated time of cross-over of their center, but are blinded to the randomization sequence for all other centers. Blinding of treatment strategy for clinicians or study personnel is not feasible due to the study design. Primary outcomes will be assessed by individual members of an adjudication committee blinded to the intervention (i.e. whether this patient was treated before or after implementation of the algorithm). Patients will be coded by a numeric randomization key, and only the principal investigators have access to this key.

### Statistical analysis

For statistical analysis, outcomes of all patients undergoing pancreatic resection before implementation of the algorithm (i.e. current practice) will be compared to outcomes of all patients undergoing pancreatic resection after implementation of the algorithm (i.e. best practice). The date of pancreatic resection will determine the study phase in which a patient will be analyzed. The primary analysis will be on an intention-to-treat basis according to the planned date of cross-over to care according to the algorithm. Data from patients in the wash-in period will be excluded from the primary analysis. Secondary analyses include a per-protocol analysis in which we will compare patients who receive care according to the algorithm (i.e. all resections after first implementation presentation at a center, including patients undergoing pancreatic resection in the wash-in period) to patients undergoing pancreatic resection before cross-over. When appropriate, 95% confidence intervals will be calculated. In all analyses a two-sided value of alpha of 0.05 will be used to denote statistical significance.

Missing baseline data will be imputed using multiple imputation techniques. The primary analysis will be performed using the multiple imputed data. A complete case analysis will be performed to check for inconsistencies. Baseline data will be analyzed and reported using standard descriptive statistics. The chi-square or Fisher’s exact test is used to compare categorical variables as appropriate. Parametric continuous variables are presented as mean with standard deviation (SD) and are compared using Student’s *t* test. Non-parametric continuous variables are presented as median with interquartile range (IQR) and are compared using the Mann-Whitney *U* test.

The primary endpoint (i.e. composite of post-pancreatectomy bleeding, new-onset organ failure and death) is a binary variable that will be analyzed using mixed-effects logistic regression analysis. Secondary endpoints will be analyzed using crude and adjusted mixed-effects logistic regression analysis (for binary outcomes) or mixed-effects linear regression analysis (for continuous outcomes). Crude analysis will include a random intercept and random slope at the level of the hospital to adjust for the design, and no other covariates. Adjusted analysis will additionally include the following covariates: calendar time and time since cross-over as a continuous variable, predictors for postoperative pancreatic fistula (i.e. soft pancreatic texture, small-diameter pancreatic duct, increasing blood loss during pancreatic resection and underlying disease that is *not* pancreatitis or pancreatic adenocarcinoma [33]) and predictors for the primary endpoint (i.e. male gender, increasing age, American Society of Anesthesiologists (ASA) classification > 2, index pancreatic resection pancreatoduodenectomy; based on multivariable logistic regression analysis as presented in Table 4). Relevant model assumptions (such as distribution of residuals) will be checked for all models; if deviations from the analysis plan are required this will be described. Crude and adjusted odds ratios with 95% confidence interval and *p* value will be reported.

**Table 4:** Factors associated with the composite primary endpoint in 1686 patients undergoing pancreatic resection in the Dutch Pancreatic Cancer Audit

Outcome	Univariable		Multivariable	
	OR (95% CI)	*P* value	OR (95% CI)	*P* value
Male gender	1.64 (1.20–2.23)	0.002	1.64 (1.19–2.27)	0.002
Age	1.02 (1.01–1.04)	0.003	1.02 (1.00–1.03)	0.04
BMI	1.00 (1.00–1.01)	0.52		
ECOG performance score		0.17		
2 vs. 1	1.29 (0.92–1.79)	0.14		
3 vs. 1	1.59 (0.95–2.66)	0.08		
4 vs. 1	0.44 (0.06–3.33)	0.43		
ASA classification (3 and 4 vs. 1 and 2)	1.87 (1.33–2.61)	< 0.001	1.79 (1.26–2.53)	0.001
Preoperative additional nutrition		0.05		
Oral vs. none	0.98 (0.70–1.38)	0.91		
Via nasogastric tube vs. none	1.74 (0.92–3.28)	0.90		
Via TPN vs. none	4.04 (1.17–14.02)	0.03		
Preoperative biliary drainage	0.97 (0.71–1.32)	0.82		
Distal pancreatectomy vs. pancreatoduodenectomy	0.50 (0.31–0.79)	0.003	0.58 (0.36–0.94)	0.03
Texture pancreas (hard/firm vs. soft/normal)	1.22 (0.86–1.73)	0.26		

Data from the Dutch Pancreatic Cancer Audit (2014–2015). Presented are the outcomes of univariable and multivariable logistic regression model showing gender, age, American Society of Anesthesiologists (ASA) classification and type of index resection are independently associated with the occurrence of the composite primary endpoint

*OR* odds ratio, *BMI* body mass index, *ECOG* Eastern Cooperative Oncology Group, *TPN* total parental nutrition

Length of hospital stay will be calculated from the date of the index pancreatic resection and will be analyzed using mixed-effects Cox proportional hazards regression, using discharge alive as the outcome event. Patients who die during the index hospitalization will be censored at the day of death. Length of ICU stay and time to resolution of pancreatic fistula (i.e. time to removal of the last abdominal drain or completion of pancreatectomy) will be analyzed using a zero-inflated negative binominal regression model.

### Subgroup and sensitivity analyses

Analyses will be performed both on the group of patients undergoing pancreatoduodenectomy and on the entire group of all patients undergoing pancreatic resection. Additional subgroup analyses will be performed based on adherence to the study protocol (i.e. proportion of patients’ days in which the algorithm is followed), hospital volume (> 45 vs. ≤ 45 pancreatic resections a year), pathologic nature of disease (malignant disease), in patients with postoperative pancreatic fistula and in patients with a high risk of pancreatic fistula [34].

A sensitivity analysis will be performed to evaluate the impact on outcomes related to postoperative pancreatic fistula. To this aim, the adjudication will be asked if an outcome was related to postoperative pancreatic fistula and if this outcome could have been prevented by adherence to the algorithm. Two separate analyses will be performed on the Comprehensive Complication Index: one analysis including all complications and one adjusted analysis, in which interventions imposed by the algorithm (e.g. minimal invasive catheter drainage and use of antibiotic management for abdominal sepsis) will not be included in the calculation. A sensitivity analysis will be performed to evaluate the success of implementation. Centers will be divided into equally sized clusters based on the percentage of additional abdominal CT scans per patient after implementation, as compared with before implementation. Separate analysis will be performed for each group.

### Economic evaluation

A cost-effectiveness analysis will be performed to compare the health effects and costs of treatment according to the algorithm, as compared with current practice. Preventing major complications is likely to be cost saving, for example, by preventing admission to the ICU and decreasing the length of hospital stay. However, additional costs may also be introduced by increasing the need for diagnostic resources (e.g. biochemical tests and CT scans) and possibly also minimally invasive interventions. Therefore, it is important to assess whether these costs will be counterbalanced by future health effects and cost savings.

For calculation of cost-effectiveness from a healthcare perspective, the volume of healthcare consumption will be measured during the trial using an adapted version of the Medical Consumption Questionnaire (iMCQ). This questionnaire will measure healthcare utilization for medical expenses both in hospital and out of hospital, including but not limited to medication, invasive interventions, days in the hospital, outpatient visits after discharge and visits to the general practitioner. Unit costs will be derived from tariffs as described in the “Zorginstituut Nederland Kostenhandleiding” [35]. Medication costs will be derived from the “Zorginstituut Nederland Medicijnkosten” and, if dosages are missing in the iMCQ, standard dosages will be derived from the “Zorginstituut Nederland Farmacotherapeutisch Kompas”. For calculation of cost-effectiveness from a societal perspective, the Productivity Consumption Questionnaire (iPCQ) will be used to derive losses in productivity. Travel costs per individual will be calculated using average travel distances and standard tariffs from the Zorginstituut Nederland Kostenhandleiding in combination with the number of visits in the iMCQ. Health-related quality of life will be measured using the Euroqol 5 dimensions-5 levels questionnaire (EQ5D-5 L) at baseline and 90 days after pancreatic resection (i.e. end of follow up) [36]. Relevant outcomes will be extracted from case record forms and electronic health records at the end of follow up. The quality-of-life scores related to these outcomes will be derived from scientific literature for calculation of cost-effectiveness. Quality-adjusted life years (QALYs) will be calculated by multiplying the duration of time spent in a health state by the corresponding quality-of-life score, hence combining life years and quality of life.

Trial-based cost-effectiveness analysis (CEA) will be performed using the empirical data comparing current practice to best practice up to 90 days after the index pancreatic resection [37]. Missing data on either health effects or costs will be imputed using multiple imputation. An additional model-based CEA will be performed to extrapolate the trial results beyond the trial duration [38–40], because the short-term effects from implementation of the algorithm - if present - are likely to affect long-term outcomes as well. Besides having influence on the primary outcome, implementation of best practice might also influence the risk of pancreatic insufficiency and possibly survival, as patients are in a better clinical condition to receive adjuvant chemotherapy. Using mathematical modeling, short-term evidence from the PORSCH trial will be translated to more generic outcomes, such as survival and health status. Finally, using the mathematical model and additional evidence from the literature, a cost-effectiveness analysis will be performed calculating the long-term additional costs and QALYs when implementing the algorithm for a lifetime horizon.

The incremental cost effectiveness ratio (ICER) will be calculated for both the trial-based and model-based CEA by dividing the difference in costs between usual care and the intervention by the difference in QALYs between usual care and the intervention. Sensitivity analysis will be performed on parameters that are expected to have the largest uncertainty. Bootstrapping and probabilistic sensitivity analysis will be performed in the trial-based and model-based CEA, respectively, to determine the uncertainty surrounding incremental health effects and costs. Results from these uncertainty analyses will be used to create (incremental) cost-effectiveness planes to graphically represent these results [40]. Cost-effectiveness acceptability curves will be calculated to demonstrate the probability that the intervention strategy will be cost-effective compared to current practice when using a range of cost-effectiveness thresholds (i.e. the amount society is willing to pay for an additional QALY).

## Discussion

In this study protocol we describe a nationwide, stepped-wedge, cluster-randomized trial on the implementation of an algorithm focusing on early detection and minimally invasive management of pancreatic fistula after pancreatic resection. By implementation of this algorithm, we expect a reduction in major complications (i.e. bleeding and organ failure) and death, as compared with current practice.

Several other studies aimed to improve the outcomes by prevention or prediction of postoperative pancreatic fistula [34, 41–43]. Although these studies have led to an improved quality of care worldwide, pancreatic fistula still remains one of the most feared complications after pancreatic surgery. When not recognized early, pancreatic fistula may have a major impact on the clinical course and is associated with an increased risk of in-hospital death, prolonged in-hospital stay, lower chance of receiving adjuvant chemotherapy and impaired long-term survival [3, 5, 1]. A recent study showed that the difference in clinical outcome in Dutch centers can be explained by a difference in the failure-to-rescue rate rather than the incidence of major complications [11]. In the Netherlands, pancreatic surgery is centralized in high-volume centers (i.e. > 20 pancreatoduodenectomies annually). Study initiatives are coordinated by the Dutch Pancreatic Cancer Group, a multidisciplinary collaboration aiming to improve the quality of care for patients with pancreatic cancer in The Netherlands, including this study. The stepped-wedge, cluster-randomized trial is a relatively new concept to evaluate the implementation of best-practice interventions at a multicenter level; several studies have had positive results [16, 44]. When implemented nationally, this trial might improve clinical outcomes at a nationwide level, and not only for selected patients in high-volume centers.

Leakage of pancreatic enzymes causes a local inflammatory response and thereby excretion of acute-phase enzymes like C-reactive protein, release of white blood cells and clinical signs of inflammation like fever, tachycardia and tachypnea [45, 46]. Amylase levels might be elevated when measured in abdominal drain fluid [10]. The abdominal drain might, however, not be in an adequate position to drain pancreatic leakage. In addition, abdominal drains are becoming more frequently omitted at the time of pancreatic resection or are removed in the early postoperative phase [47]. In these patients, signs of inflammation are the only early signs of postoperative pancreatic fistula. These signs are, however, nonspecific and often not recognized as potential pancreatic leakage, especially in centers where only a few patients with pancreatic fistula are seen annually [45]. We believe, however, that in these patients with a high risk of life-threatening complications, early abdominal CT should be performed and peripancreatic fluid collections should be managed aggressively with percutaneous drainage in patients showing signs of systemic inflammation [9, 48, 49]. This might result in an increased use of abdominal imaging and percutaneous catheter drainage procedures in the entire study population, with the intention of reducing severe bleeding, organ failure or mortality in a subset of patients. The outcomes of this trail will provide more insight into the “number needed to scan” or “number needed to drain” and the cost-effectiveness of intensified postoperative monitoring.

The management of pancreatic fistula after pancreatic resection is shifting from relaparotomy to a more conservative approach using minimally invasive catheter drainage. Pancreatic fistula after pancreatoduodenectomy leads to an intra-abdominal fluid collection filled with activated pancreatic juices. When drained adequately, even a severe pancreatic fistula could resolve with primary catheter drainage alone [9]. In addition, percutaneous drainage is a minimally invasive procedure, which will provoke less surgical trauma (i.e. tissue injury and systemic inflammatory response) compared with relaparotomy. Even moderately small surgical trauma that induces a proinflammatory cytokine response can lead to organ failure in severely ill patients [12]. There is, however, still a significant number of patients in whom pancreatic fistula is managed primarily through laparotomy. Clinical outcomes might be improved using a step-up approach with primary catheter drainage and relaparotomy as a salvage option in patients who continue to deteriorate without further options for minimally invasive drainage.

This trial has several limitations. Even though a stepped-wedge, cluster-randomized trial is less efficient as compared with a conventional individual randomized trial, this is the only appropriate design to evaluate our research question, which is based on education in “best practice care” involving early detection and management of pancreatic fistula. With a design based on individual-level randomization, contamination of the control group would have been very likely due to the learning effect of applying the algorithm in the intervention group. The more efficient, cluster-randomized, cross-over design, in which half of the clusters crosses over from control to intervention and the other from intervention to control, is also prohibited due to the learning effect. Moreover, a stepped-wedge design was also chosen for logistical reasons, i.e. the aim is to implement the algorithm in all Dutch pancreatic centers and it is not feasible to roll out an education-based intervention with very active participation of the study team in more than one center simultaneously. Additionally, after calculating the statistical efficiency, the power achieved from a stepped-wedge, cluster-randomized trial was considerably higher than that of a parallel cluster-randomized trial, which is due to relatively high intra-cluster correlation.

Still, there is a risk that current practice will be contaminated by the proposed intervention in this study, which would result in a bias towards no effect. To truly evaluate the effect of the implementation of the algorithm it is essential to minimize the risk of contamination in the centers where patients are still being treated according to current practice. If all clinicians were intensively involved in the development of the algorithm, they might adjust their current practice to the algorithm before the start of the trial. On the other hand, for optimal implementation it is essential that the centers feel involved in the development of the intervention. Therefore, the algorithm was designed by a group of stakeholders of the Dutch Pancreatic Cancer Group. The proposed algorithm was validated in a retrospective multicenter cohort, as prospective implementation would have increased the risk of contamination. Next, the algorithm was discussed at a meeting with one leading surgeon with expertise in pancreatic surgery from every participating center, using a PowerPoint presentation, the content of which was not shared with the audience electronically or on paper. At this meeting, consensus was reached on the content of the algorithm. The pancreatic surgeons were instructed not to share with their colleagues what was discussed during the meeting. The algorithm was not distributed within the centers prior to the start of the wash-in period. Consensus was reached on the final algorithm with internationally respected experts in the field of pancreatic surgery before nationwide implementation in this trial. We believe this approach will ensure local support for the intervention while minimizing potential bias through knowledge of its components.

In conclusion, we have designed a stepped-wedge, cluster-randomized trial to evaluate the effect of the nationwide implementation of an algorithm for early detection and minimally invasive management of pancreatic fistula after pancreatic resection on severe complications and mortality in The Netherlands.

## Trial status

This manuscript is based on the PORSCH study protocol version 7, December 2017. The Medical Ethical Committees United (MEC-U) approved the study and waived the need for informed consent (reference number W17.057). Local approval for the study is obtained in all participating centers before recruitment starts.

First inclusion: 8 January 2018.

Interim analysis: 8 November 2018, 569 patients included. Conclusion: enrollment as planned, trial continues without adaptation.

Planned last inclusion: 9 November 2019.

Trial status at time of submission: recruiting.

## Supplementary information

**Additional file 1: Appendix 1.** Design of the Algorithm. **Appendix 2.** Interpretation And Rationale of the Algorithm.

## Data Availability

Study results will be presented at (inter)national meetings and published in a peer-reviewed journal. The datasets used and/or analyzed during the current study are available from the corresponding author on reasonable request.

## Abbreviations

CCI: Comprehensive complication index
CEA: Cost-effectiveness analysis
CRP: C-reactive protein
CT: Computed tomography
DPCA: Dutch Pancreatic Cancer Audit
ICC: Intra-cluster correlation
ICER: Incremental cost effectiveness ratio
ICU: intensive care unit
iMCQ: Medical Consumption Questionnaire
iPCQ: Productivity Consumption Questionnaire
IQR: Interquartile range
MEC-U: Medical Ethical Committees United
MODS: Multiple organ dysfunction score
POD: Postoperative day
PORSCH: Postoperative standardization of care, the implementation of best practice after pancreatic resection
QALYs: Quality-adjusted life years
SD: Standard deviation
SIRS: Systemic inflammatory response syndrome
SOFA: Sequential organ failure assessment
WBC: White blood cell count
