# Supplementary material for: Care after pancreatic resection according to an algorithm for early detection and minimally invasive management of pancreatic fistula versus current practice (PORSCH-trial): design and rationale of a nationwide stepped-wedge cluster-randomized trial
Source: Trials. 2020 May 7;21:389. doi: 10.1186/s13063-020-4167-9 (PMC7206814; doi:10.1186/s13063-020-4167-9)
Supplement: Supplementary file 1 — Additional file 1: Appendix 1. Design of the Algorithm. Appendix 2. Interpretation And Rationale of the Algorithm. [file 13063_2020_4167_MOESM1_ESM.docx]

**APPENDIX 1: Design of the Algorithm**

A schematic overview of the construction is provided in the figure below.


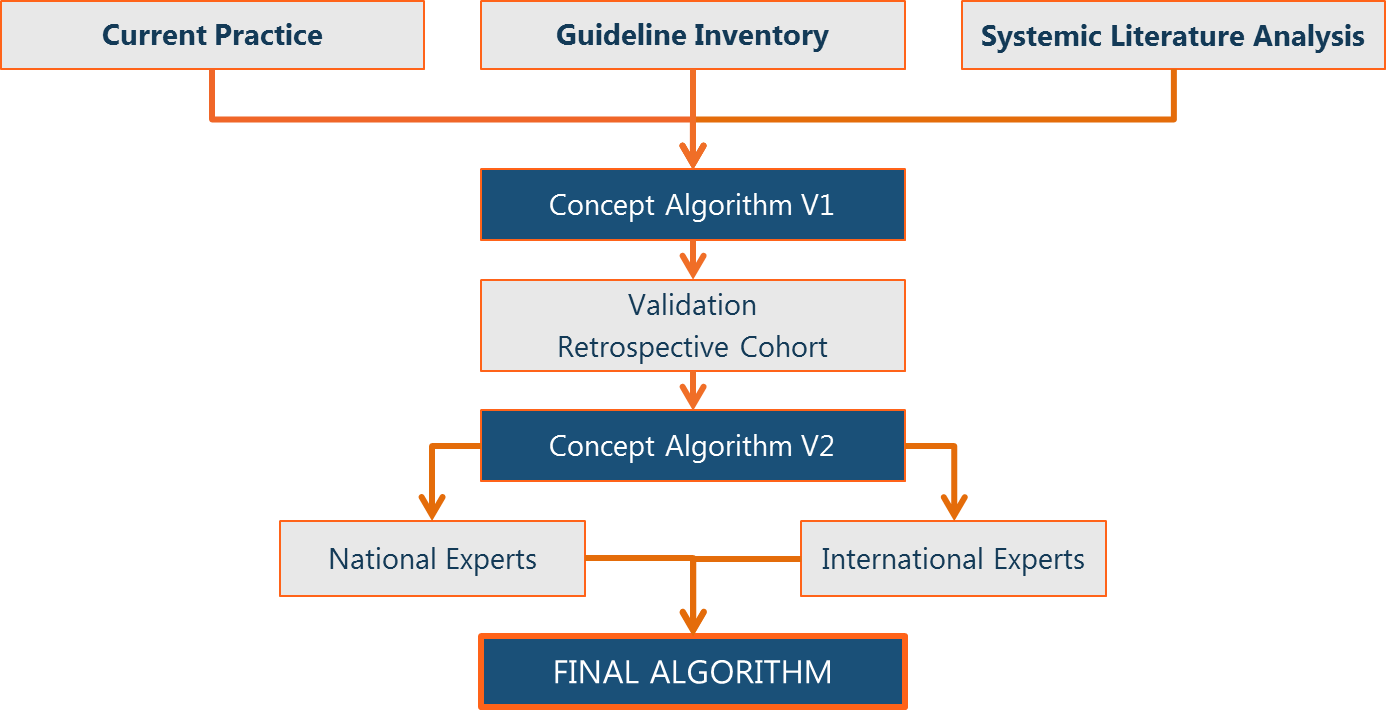


**Current practice**

The rationale for this study is underlined by two recent analyses in Dutch data. They also provide the base for the design of the algorithm and will therefore be discussed first.

*Failure to rescue study*

Mortality in patients with major complications (i.e. failure to rescue) was evaluated in over 1300 consecutive patients undergoing pancreatoduodenectomy in 2014 and 2015. Data were obtained in the mandatory Dutch Pancreatic Cancer Audit. The at the time 18 hospitals performing pancreatic surgery in The Netherlands were divided into quartiles based on in-hospital mortality. Mortality was 0.9% in the first hospital quartile (327 patients in 4 hospitals) and 8.1% in the fourth quartile (310 patients in 5 hospitals). Incidence of major complications (i.e. Clavien-Dindo grade III and higher) and mortality were compared between these quartiles. Hospitals with a high mortality rate had a slightly increased major complication rate (40% increase), but a much higher failure to rescue rate (560% increase), as compared with hospitals with a low mortality rate. This strongly suggest that complication management is the most important factor in improving clinical outcomes included lowering postoperative mortality.

*Management of postoperative pancreatic fistula*

Severe pancreatic fistula is defined as leakage of pancreatic enzymes into the abdominal cavity after pancreatic resection requiring invasive intervention. Traditionally this is done by a relaparotomy during which the abdomen is re-opened, the intra-abdominal cavity is flushed and the leaking pancreatic anastomosis can be handled in different ways. An alternative treatment in the form of image-guided catheter drainage is gaining popularity. The advantage of the percutaneous catheter drainage is the minimally invasive character, as compared with relaparotomy. This means the already critically ill patient does not have to suffer from pro-inflammatory stress induced by a major reoperation. This management strategy could thereby lead to a reduction in in multiple organ failure typically seen in these patients.

We performed a retrospective, propensity-score matched cohort study in 9 centers of the Dutch Pancreatic Cancer Group to investigate the superiority of catheter drainage over relaparotomy. Propensity score matching was used to limit the effect of confounding by indication. The primary endpoint was death, secondary endpoints included new-onset organ failure.

From a cohort of 2196 consecutive pancreatoduodenectomies, 309 patients with severe pancreatic fistula were included. Overall in-hospital mortality was 18% (55/309 patients). Overall, 227 patients (73%) underwent primary catheter drainage and 82 patients (27%) underwent primary relaparotomy. Primary catheter drainage was successful (i.e. survival without relaparotomy) in 77% of patients. With propensity-score matching, 64 patients undergoing primary relaparotomy were matched to 64 patients undergoing primary catheter drainage. Mortality was lower after catheter drainage (14% vs. 36%; P=0.007; risk ratio 0.39; 95% confidence interval 0.20-0.75). The rate of new-onset single-organ failure (3% vs. 20%; P=0.007; risk ratio 0.15; 95% confidence interval 0.03-0.60), and new-onset multiple-organ failure (16% vs. 39%; P=0.008; risk ratio 0.40; 95% confidence interval 0.20-0.77) were also lower after primary catheter drainage. Comparison of management strategies between the participating centers showed a range of 12% to 67% of patients undergoing relaparotomy as primary intervention for pancreatic fistula.

In conclusion, this propensity-matched study showed primary relaparotomy is associated with twofold higher mortality, as compared with primary catheter drainage as first intervention for pancreatic fistula. This study showed as well that uniformity in the management of pancreatic fistula is currently lacking between the different centers in the Netherlands. Many centers still perform primary relaparotomy, which was clearly found to be associated with higher rates of multiple-organ failure and death, as compared to minimally invasive catheter drainage.

**Guideline inventory**

To objectivity the current level of standardization in care after pancreatic resection, an inventory in local guidelines was performed. This inventory showed a considerable number of centers reported regular measurement of vital parameters, CRP and WBC, but only one center reported standardized evaluation of these measurements (i.e. use of predefined cut-offs; shown as gray dots in table 1).

In conclusion, none of the 12 evaluated guidelines clearly stated how to manage postoperative pancreatic fistula. Outcomes of this inventory are presented in the table below.

| **Table 1: inventory local guidelines** | | | | | | | | | |
| --- | --- | --- | --- | --- | --- | --- | --- | --- | --- |
| **CENTER** | **Vital parameters** | **CRP** | **WBC** | **Consult**  **Pancreatic Surgeon** | **Predefined Indication**  **CT Scan** | **Predefined Indication Invasive Intervention** | **Management**  **Pancreatic Fistula** | **Drain Amylase** | **Removal Of**  **Abdominal Drain** |
| **1** | ● | ● | ○ | ○ | ● | ○ | ○ | ● | ● |
| **2** | ● | ○ | ○ | ○ | ○ | ○ | ○ | ○ | ○ |
| **3** | ○ | ○ | ○ | ○ | ○ | ○ | ● | ○ | ● |
| **4** | ○ | ● | ● | ○ | ○ | ○ | ○ | ● | ● |
| **5** | ○ | ● | ● | ○ | ○ | ● | ● | ● | ● |
| **6** | ● | ● | ● | ○ | ○ | ○ | ○ | ● | ● |
| **7** | ● | ○ | ○ | ○ | ○ | ○ | ○ | ● | ● |
| **8** | ○ | ○ | ○ | ○ | ○ | ○ | ○ | ○ | ● |
| **9** | ● | ● | ● | ○ | ○ | ○ | ○ | ● | ● |
| **10** | ○ | ○ | ○ | ○ | ○ | ○ | ○ | ○ | ● |
| **11** | ○ | ● | ● | ○ | ○ | ○ | ● | ● | ○ |
| **12** | ○ | ● | ● | ○ | ○ | ○ | ○ | ● | ● |
| *Table on inventory of local guidelines in 12 centers of the DPCG;* ●*, variable well defined in local protocol;* ●*, measurement of variable defined in local protocol, but no specification on evaluation;* ○*, variable not defined in local protocol. Abbreviations: CRP, C-reactive protein; WBC, white blood count; CT, Computed Tomography* | | | | | | | | | |

**Systematic literature analysis**

As described above, the major challenge to improve outcome of patients undergoing pancreatic resection is to treat pancreatic fistula before it leads to severe clinical deterioration with systemic complications. This means diagnostic modalities should be identified that have high accuracy in detecting pancreatic fistula at an early stage. To this end, we have performed extensive literature analysis to evaluate the accuracy of physical examination parameters, biochemical test and imaging modalities for diagnosing severe postoperative pancreatic fistula. Results of this analysis have been published recently[1].

**Concept algorithm**

Results from these projects were combined into the first version of the concept algorithm. Parameters and cut-offs included in this algorithm are extracted from the systematic literature analysis and from the abovementioned Dutch studies. This first concept version of the algorithm was discussed with a small group of stakeholders within the Dutch Pancreatic Cancer Group to evaluate the clinical applicability of the proposed algorithm. Decisions on how to adjust the proposed algorithm were made during several consensus meetings.

**Validation retrospective cohort**

The effect in terms of number and timing of abdominal CT scans was evaluated in a retrospective cohort. Because of the risk of contamination (i.e. health care providers changing their current practice after reviewing the algorithm), we could not perform a formal prospective pilot study. With this validation study we aimed to evaluate the performance of the algorithm.

All patients undergoing pancreatoduodenectomy or distal pancreatectomy in 2016 in 3 centers of the Dutch Pancreatic Cancer Group were included. Data on patient characteristics, pancreatic resection, postoperative parameters (i.e. body temperature, respiratory rate, heart rate, CRP, WBC, drain amylase) and details on hospitalization course including timing of performed CT scans and invasive interventions were collected. To evaluate the ability of the algorithm to those select patients undergoing a CT scan on the same day or one day before that day in reality, diagnostic outcomes were calculated. Secondary outcomes were total number of CT scans, timing of first CT scan and invasive interventions.

A total of 174 patients were included (53% male, median age 67 years (inter quartile range 57-73); 133 pancreatoduodenectomies and 41 distal pancreatectomies). The algorithm revealed an accuracy of 73%, positive predictive value 15% and negative predictive value 96%. A total 65 patients underwent an abdominal CT scan according to current practice, whereas a total of 85 patients woud have undergone a CT scan according to the algorithm. 22 out of 23 patients with severe pancreatic fistula (i.e. requiring invasive intervention) underwent a CT scan according to the algorithm. In the patient not appointed to a CT scan according to the algorithm, the majority of clinical data was missing. The median timing of first CT scan was postoperative day 5 in current practice (interquartile range 3 to 7). This would have been postoperative day 3 according to the algorithm (interquartile range 3 to 4). A total of 36 patients underwent percutaneous drainage after pancreatic resection, this was performed on postoperative day 3 in 5 patients. In addition, 2 patients underwent percutaneous drainage on postoperative day 4 based on an abdominal CT scan performed on postoperative day 3.

In summary, the algorithm resulted in more scans in the early postoperative phase. However, the overall increase in CT scans is expected to be moderate for early detection of pancreatic fistula will prevent a severely complicated postoperative course. Starting daily clinical evaluation on postoperative day 3 appears to be sufficient, as in 20% of patients ultimately undergoing percutaneous drainage this was indicated based on a CT scan performed on postoperative day 3. In conclusion, the algorithm appears to be effective in early detection of those patients ultimately developing severe pancreatic fistula.

**Expert opinion**

The proposed and validated algorithm was discussed at a meeting with one pancreatic surgeon from every center of the Dutch Pancreatic Cancer group. Only one (mostly the leading pancreatic surgeon) was invited from each center to prevent the algorithm to become known to a larger group of specialists involved in the care of patients with pancreatic cancer, to prevent contamination during the pre-implementation phase of the study. Suggestions for improvement were made after which consensus was reached on both the algorithm and study design.

Three internationally highly respected experts in the field of pancreatic surgery were asked to serve in an advisory committee. These experts are asked to critically review the content of the algorithm and study design during different phases of the project.

**APPENDIX 2: Interpretation And Rationale of the Algorithm**

The interpretation and rationale for the construction of this algorithm, including proposed exact cut-offs and thresholds are explained in this document. All parameters included in the algorithm are:

1. Associated with early detection or improved outcome in patients with clinically relevant pancreatic fistula as demonstrated in the literature or
2. Widely used in current practice for early detection or generally associated with an improved outcome in patients with clinically relevant pancreatic fistula

The best practice algorithm was discussed at meeting with the study steering committee and the leading pancreatic surgeon from every Dutch center performing pancreatic surgery and with the leading pancreatic surgeons from three internationally renowned centers (i.e. *Johns Hopkins Medical Center Baltimore, UPMC Pittsburgh, Karolinska Institutet Stockholm*). There is consensus amongst all experts involved in this trial that this algorithm represents the best quality of postoperative care for patients undergoing pancreatic resection.

**
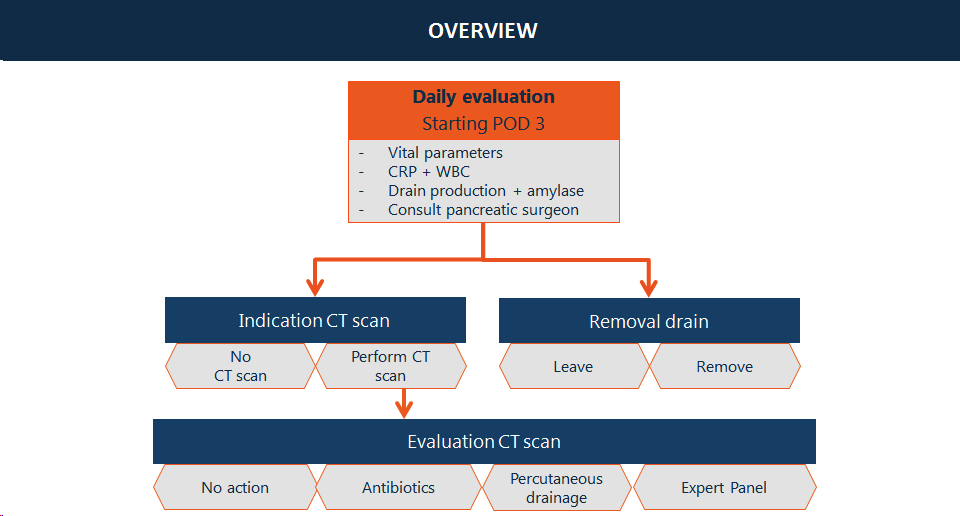
**

*Daily evaluation starting postoperative day 3*

The algorithm for early detection of pancreatic fistula starts on the third postoperative day. Pancreatic fistula only rarely occurs within the first 3 days[2] and physiologic postoperative inflammatory response limits the distinctiveness of inflammatory markers in the early postoperative phase[3].

**
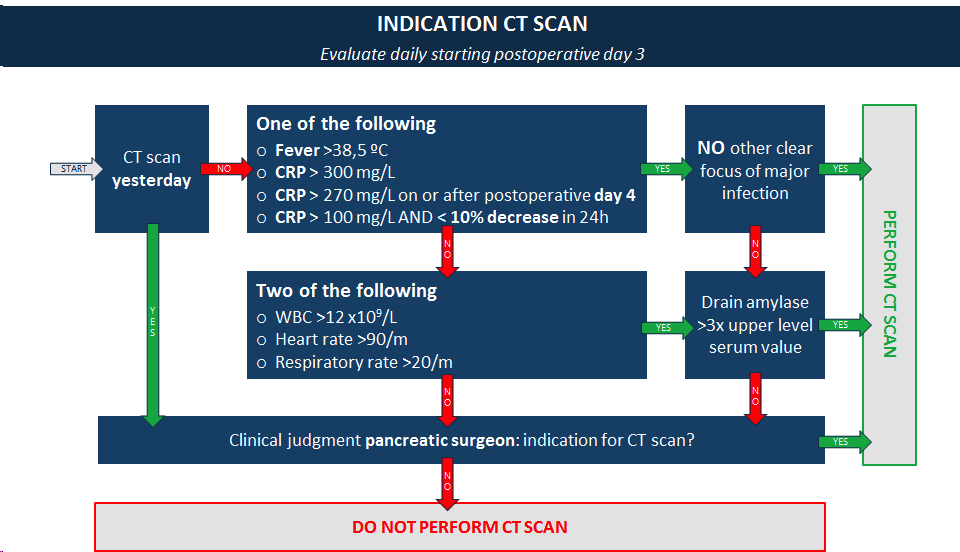
**

**General considerations when evaluating the indication for abdominal CT scan**

- Abdominal CT scan on two consecutive days is only indicated based on the clinical judgment of the pancreatic surgeon
- The indication for abdominal CT scan is based on abnormalities in inflammatory parameters
- The local pancreatic surgeon is asked to evaluate if there is another clear focus of major infection might cause these inflammatory abnormalities, bearing in mind that other infections (e.g. pneumonia or wound infection) could be secondary to an abdominal focus.
- An abdominal CT scan is always indicated in patients with abnormalities in inflammatory parameters in combination with an elevation of drain amylase level
- One local pancreatic surgeon should visit the admitted postpancreatectomy patients daily. For practical reasons, in weekdays (Monday to Friday) this should by in person, in weekends (Saturday and Sunday) they should (at least) personally discuss the patient with the attending gastrointestinal surgeon or resident

*CT scan yesterday*

A safety measure was introduced in to prevent patients from undergoing daily CT scans. Therefore, CT scans on two subsequent days are only indicated if the pancreatic surgeon sees any reason to do so.

*Fever*

Temperature was included in the algorithm based on the current literature and expert opinion. The association between elevation in body temperature after pancreatic resection and severe postoperative pancreatic fistula was evaluated in 3 studies on a total of 435 patients[4–6]. All outcomes of these studies are provided in the table below.

| **Table 1: Outcomes on diagnostic accuracy of temperature for severe pancreatic fistula** | | | | | | | | | |
| --- | --- | --- | --- | --- | --- | --- | --- | --- | --- |
| **Reference** | **N** | **Severe PF** | **Cut-off** | **Day** | **Sens.** | **Spec.** | **PPV** | **NPV** | **Accuracy** |
| Noji 2011[4] | 58 | 32 | 38.0 ºC | 3 | 53 | 77 | 74 | 57 | 64 |
| Uemura 2014[6] | 200 | 15 | 37.8 ºC | 3 | 60 | 79 | 19 | 96 | 75 |
|  |  |  | 37.7 ºC | 4 | 53 | 88 | 37 | 96 | 86 |
| Moskovic 2010[5] | 177 | 17 | 38.6 ºC | Any | 18 | 97 | 50 | 88 | 86 |
| *N, total number of patients included in analysis; severe PF, severe pancreatic fistula (i.e. ISGPS grade B/C); Day, postoperative day; sens., sensitivity; spec., specificity; PPV, positive predictive value; NPV, negative predictive value; Accuracy was calculated by dividing number of correct assessments by total number of assessments. The study by Uemura et al.*[6] *evaluated two cut off points on different postoperative days.* | | | | | | | | | |

For the heterogeneity of proposed cut-offs, outcomes were not pooled. The decision on cut-off included in the algorithm was a combination of the upper limit as reported in the literature, in which a temperature of >38.5ºC is considered fever.

*C-Reactive Protein [CRP]*

The association between elevation in CRP levels after pancreatic resection and severe postoperative pancreatic fistula was evaluated in 8 studies on a total of 1605 patients[6–13]. Proposed cut-offs are spread widely, all outcomes of these studies are provided in the table below.

| **Table 2: Outcomes on diagnostic accuracy of CRP for severe pancreatic fistula** | | | | | | | | | |
| --- | --- | --- | --- | --- | --- | --- | --- | --- | --- |
| **Reference** | **N** | **Severe PF** | **Cut-off*** | **Day** | **Sens.** | **Spec.** | **PPV** | **NPV** | **Accuracy** |
| Giardino 2016[7] | 84 | 18 | 92 | 1 | 89 | 58 | 36 | 95 | 64 |
| Fujiwara 2013[10] | 297 | 166 | 94 | 1 | 65 | 60 | 67 | 57 | 62 |
| Palani Velu 2016[11] | 185 | 43 | 180 | 2 | 83 | 36 | 29 | 88 | 47 |
| Furukawa 2016[10] | 46 | 16 | 100 | 3 | 59 | 88 | 81 | 70 | 74 |
| Ansorge 2014[12] | 315 | 59 | 202 | 3 | 78 | 83 | 51 | 94 | 82 |
| Solaini 2015[14] | 378 | 31 | 272 | 3 | 50 | 77 | 17 | 94 | 74 |
| Uemura 2014[6] | 200 | 15 | 145 | 3 | 100 | 53 | 15 | 100 | 80 |
|  |  |  | 156 | 4 | 80 | 87 | 22 | 98 | 80 |
| Kosaka 2014[8] | 100 | 34 | 93 | 4 | 88 | 90 | 90 | 94 | 80 |
| *N, total number of patients included in analysis; severe PF, severe pancreatic fistula (i.e. ISGPS grade B/C); Day, postoperative day; sens., sensitivity; spec., specificity; PPV, positive predictive value; NPV, negative predictive value; Accuracy was calculated by dividing number of correct assessments by total number of assessments. The study by Uemura et al.*[6] *evaluated two cut off points on different postoperative days.*  ** Values in milligram per liter* | | | | | | | | | |

In the algorithm were included the lower and upper border of proposed cut-offs (i.e. 100 to 270 for practical reasons). Evaluation in the retrospective cohort showed a high number of patients (53 of 153 patients) having a CRP level over 270 mg/L on the third postoperative day, with a high false positive rate. Therefore, in consensus among expert Dutch pancreatic surgeons, the cut-off level was increased to 300 mg/L only for the third postoperative day.

*Systemic Inflammatory Response Syndrome [SIRS]*

The association between elevation in white blood cell count (WBC) after pancreatic resection and severe postoperative pancreatic fistula was evaluated in 3 studies on a total of 358 patients[4,6,8]. All outcomes of these studies are provided in the table below.

| **Table 3: Outcomes on diagnostic accuracy of WBC for severe pancreatic fistula** | | | | | | | | | |
| --- | --- | --- | --- | --- | --- | --- | --- | --- | --- |
| **Reference** | **N** | **Severe PF** | **Cut-off*** | **Day** | **Sens** | **Spec** | **PPV** | **NPV** | **Accuracy** |
| Noji 2012[4] | 58 | 32 | 9.7 | 3 | 91 | 62 | 74 | 84 | 78 |
| Uemura 2014[6] | 200 | 15 | 9.8 | 3 | 87 | 63 | 16 | 98 | 65 |
|  |  |  | 9.3 | 4 | 73 | 82 | 25 | 97 | 82 |
| Kosaka 2014[8] | 100 | 32 | 7.4 | 4 | 81 | 71 | 57 | 89 | 90 |
| *N, total number of patients included in analysis; severe PF, severe pancreatic fistula (i.e. ISGPS grade B/C); Day, postoperative day; sens, sensitivity; spec, specificity; PPV, positive predictive value; NPV, negative predictive value; Accuracy was calculated by dividing number of correct assessments by total number of assessments. The study by Uemura et al.*[6] *evaluated two cut off points on different postoperative days.*  ** Values in cells x10^9^ per liter* | | | | | | | | | |

The proposed cut-offs are all relatively low and sometimes within normal ranges of WBC. Therefore, we chose to combine WBC with other two SIRS criteria (i.e. heart rate >90/min and respiratory rate >20/min for body temperature is evaluated as separate variable). Even though there is no evidence showing the direct relation between SIRS and severe pancreatic fistula, SIRS is widely accepted as an early marker for sepsis and includes readily available parameters[15]. SIRS cut-off points were included in the proposed algorithm.

*NO other clear focus of major infection*

An abdominal CT scan is indicated if CRP levels are raised above the proposed thresholds (i.e. >300; >270 or >100 without significant improvement) and there is no other very clear focus for major infection that could explain the deviation from normal value. The decision on whether another focus is reason enough not to perform an abdominal CT scan is left to the local pancreatic surgeon, who should be reminded that pulmonary pathology could be secondary to abdominal problems. If drain amylase is elevated, an abdominal CT scan should be performed even if another focus for infection is suspected.

*Clinical judgment pancreatic surgeon*

There are some clinical signs that cannot be categorized and are not included in this algorithm. Therefore, daily evaluation by a pancreatic surgeon is required to evaluate is there is any other indication for abdominal CT scan.

**
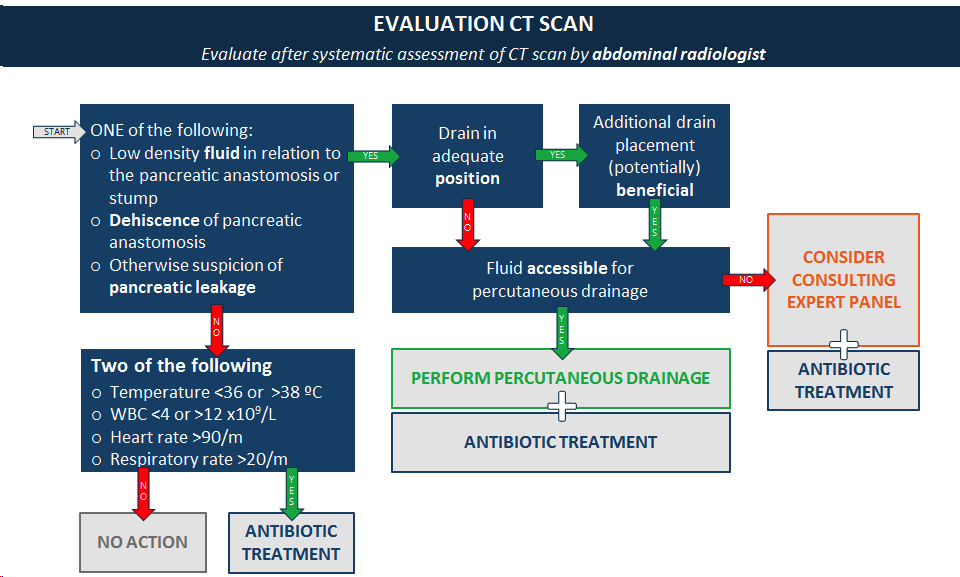
**

**General considerations when evaluating an abdominal CT scan**

- All CT scans are assessed systematically by a dedicated abdominal radiologist according to the predefined form (see APPENDIX 4 included in the study protocol)
- This algorithm should be used to evaluate the indication for (invasive) intervention for pancreatic fistula. This decision should be made in consensus with the pancreatic surgeon, radiologist and interventional radiologists
- Pancreatic fistula should preferably be managed through minimally invasive percutaneous drainage. Relaparotomy should only be considered in patients with ongoing clinical deterioration with no options for minimally invasive interventions
- A 24/7, online, nationwide, multidisciplinary expert panel is available to aid difficult clinical decisions on indication and feasibility of invasive interventions
- Management of other complications (i.e. not being pancreatic fistula) are not within the scope of this algorithm and are therefore left to discretion of the local clinicians

*Suspicion of pancreatic fistula*

The suspicion of severe pancreatic fistula is based both on clinical and radiological parameters[16,17]. Clinical suspicion is raised by abnormalities in inflammatory parameters and was evaluated in the previously discussed algorithm on indication for abdominal CT scan. The abdominal radiologists will be asked whether pancreatic fistula is suspected based on the systematic assessment of the abdominal CT scan. Suspicion is generally raised if low density fluid is seen near the transection site or anastomosis of the pancreas (i.e. not being hematoma based on density). If there is no radiologic suspicion of pancreatic fistula, there is no indication for invasive intervention for pancreatic fistula. Management of other complications that are diagnosed on abdominal CT scan is not within the scope of this algorithm and is therefore left to the discretion of local clinicians.

*Adequate drain position and (additional) drain placement*

The possibility of (additional) minimally invasive percutaneous drainage should be considered in all patients with (suspected) severe pancreatic fistula (i.e. based on clinical and radiological parameters). In patients with abdominal drain in adequate position, it should be evaluated whether additional drain placement is potentially beneficial. All fluid in the upper abdomen in patients with suspected pancreatic fistula should be evaluated for the possibility of (additional) percutaneous drainage[18–21]. If drainage is feasible, this should be the primary management of choice. Previous studies have shown that fluid should not necessarily be collected, but should be large enough to fit a 8 French pigtail drain[22,23].

*Expert panel*

An online, nationwide, multidisciplinary expert panel will be available 24/7 to aid difficult clinical decisions on indication and feasibility of invasive interventions. This concept of this expert panel is based on the Dutch Pancreatitis Study Group expert panel and therefore the design of this panel is very similar to theirs[24].

This panel exists of pancreatic surgeons, dedicated abdominal radiologists and interventional radiologists with vast experience in treating patients with abdominal complications after pancreatic resection. The expert panel is consulted by filling out an online form. The consulting clinician provides anonymous patient information, including medical history, clinical course, vital and inflammatory parameters, previous interventions and selected images from the most recent CT scan. The expert form is send to the study coordinator and then forwarded to the members of the expert panel who are also notified by a text message via mobile phone. The experts independently return their advice to the study coordinator as soon as possible. Within 12 hours, the combined expert advices are forwarded to the consulting clinician[24].

The expert panel will provide an advice on if pancreatic fistula is suspected based on the CT scan. If pancreatic fistula is suspected, additional advice is provided on the technical aspects of minimally invasive percutaneous drainage, including proposed approach and material. Surgical intervention (i.e. relaparotomy) is only considered in patients with ongoing clinical deterioration with no additional options for minimally invasive intervention. In these cases, the expert panel will be asked to evaluate the indication and feasibility of surgical intervention. They will also be asked to provide an advice on type of intervention during relaparotomy (i.e. lavage and surgical drainage or completion pancreatectomy).

In case of clinical or radiological evident persisting hepaticojejunostomy leak or mixed or drirty leak, the possibility of percutaneous transhepatic cholangiography (PTC) drainage should be considered to drain both the intrahepatic bile system and the small bowel loop of anastomoses. The expert panel is also available to provide advice on indication and feasibility of PTC drain placement.

*Antibiotic treatment*

All patients with suspected pancreatic fistula should receive antibiotic treatment[25]. In addition, patients in whom no pancreatic fistula was suspected on abdominal CT scan but with 2 out of the 4 SIRS criteria should also receive antibiotic treatment.

**
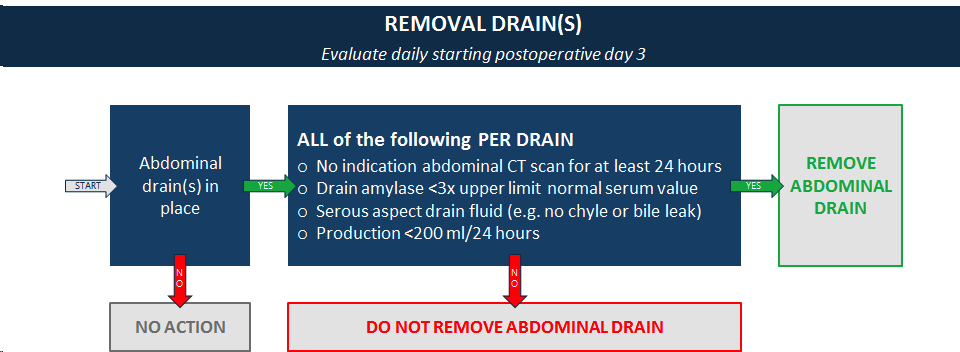
**

**General considerations when evaluating the removal of abdominal drains**

- Because abdominal drains are also a source of infection, these should be removed at the earliest possibility
- Drain fluid should be assessed for each drain separately
- Evaluation starts on postoperative day 3 for as long as there are drains in place[26]
- Patients should be in good clinical condition for at least 24 hours. As indication for abdominal CT scan was made primarily on clinical condition, these criteria were also used in this part of the algorithm

*Drain amylase level*

For early elevation in drain amylase levels are mostly used to predict pancreatic fistula, only the association between elevation in drain amylase level *on or after postoperative day 3* following pancreatic resection and severe postoperative pancreatic fistula was evaluated for the design of the algorithm. The outcomes of 9 studies[4,6,8,27–32] on a total of 1655 patients were evaluated and are provided in the table below.

| **Table 4: Outcomes on diagnostic accuracy of drain amylase on or after postoperative day 3 for severe pancreatic fistula** | | | | | | | | | |
| --- | --- | --- | --- | --- | --- | --- | --- | --- | --- |
| **Reference** | **N** | **Severe PF** | **Cut-off*** | **Day** | **Sens** | **Spec** | **PPV** | **NPV** | **Accuracy** |
| Tsujie 2012[27] | 114 | 18 | 300 | 3 | 72 | 64 | 27 | 92 | 65 |
| Dugalic 2014[28] | 382 | 57 | 800 | 3 | 90 | 99 | Nr | Nr | Nr |
| Noji 2011[4] | 58 | 32 | 3000 | 3 | 31 | 92 | 83 | 51 | 58 |
| Uemura 2014[6] | 200 | 15 | 116 | 3 | 100 | 64 | 19 | 100 | 67 |
|  |  |  | 95 | 4 | 87 | 82 | 28 | 99 | 83 |
| Kosaka 2014[8] | 100 | 32 | 647 | 4 | 72 | 91 | 79 | 87 | 85 |
| Kobayashi 2013[29] | 58 | 29 | 1000 | 3 | 69 | 22 | 74 | 18 | 58 |
|  |  |  | 1000 | 5 | 48 | 86 | 78 | 63 | 67 |
| El Nakeeb 2013[30] | 471 | 36 | 4000 | 5 | 74 | 99 | Nr | Nr | Nr |
| Shinchi 2006[31] | 207 | 17 | 500 | 5 | Nr | Nr | 45 | 100 | Nr |
| Facy 2012[32] | 65 | 14 | 1000 | Any | 79 | 78 | 50 | 93 | 79 |
| *N, total number of patients included in analysis; severe PF, severe pancreatic fistula (i.e. ISGPS grade B/C); Day, postoperative day; sens, sensitivity; spec, specificity; PPV, positive predictive value; NPV, negative predictive value; nr, not reported. Accuracy was calculated by dividing number of correct assessments by total number of assessments. The study by Uemura et al.*[6] *and Kobayashi et al.*[29] *both evaluated two cut off points on different postoperative days.*  ** Values in units per liter* | | | | | | | | | |

The proposed cut-offs are spread widely and the diagnostic outcomes differ between these studies. For practical reasons, we chose to adapt the definition as reported by the International Study Group on Pancreatic Surgery in the algorithm (i.e. <3x upper limit of serum amylase). This reflects one of the lowest cut-offs reported in the literature.

*Drain fluid appearance*

Even though chyle leak, hepaticojejunostomy leakage and gastroenterostomy leakage can be confirmed by biochemical tests, the suspicion of these complications is generally based on the appearance of the drain fluid. To prevent unnecessary additional biochemical tests, routine laboratory tests for these complications were not included in the algorithm. The criterion of ‘clear serous aspect’ was included in the algorithm to prevent the removal of drains that should be left in place for any other reason than pancreatic fistula.

*Drain production*

The association between the volume of drain production after pancreatic resection and severe postoperative pancreatic fistula was evaluated in 2 studies on a total of 407 patients[6,31]. All outcomes of these studies are provided in the table below. The upper limit of proposed cut-offs was included in the proposed algorithm (i.e. 200cc in 24 hours).

| **Table 5: Outcomes on diagnostic accuracy of drain production for severe pancreatic fistula** | | | | | | | | | |
| --- | --- | --- | --- | --- | --- | --- | --- | --- | --- |
| **Reference** | **N** | **Severe PF** | **Cut-off*** | **Day** | **Sens** | **Spec** | **PPV** | **NPV** | **Accuracy** |
| Shinchi 2006[31] | 207 | 17 | 200 | >5 | 94 | 80 | 84 | 92 | 88 |
| Uemura 2014[6] | 200 | 15 | 180 | 3 | 7 | 76 | 2 | 91 | 71 |
|  |  |  | 55 | 4 | 67 | 75 | 18 | 97 | 74 |
| *N, total number of patients included in analysis; severe PF, severe pancreatic fistula (i.e. ISGPS grade B/C); Day, postoperative day; sens, sensitivity; spec, specificity; PPV, positive predictive value; NPV, negative predictive value; Accuracy was calculated by dividing number of correct assessments by total number of assessments. The study by Uemura et al. evaluated two cut off points on different postoperative days.*  ** Values in units per liter* | | | | | | | | | |

**Addendum: hepaticojejunostomy leakage**

In case of early hepaticojejunostomy leakage (i.e. visual bile leakage through abdominal catheter within 24 hours after surgery) primary relaparotomy with revision of hepaticojejunostomy should be considered. Clinically persisting hepaticojejunostomy leakage occurring be managed through percutaneous drainage. This can either be through percutaneous drainage of abdominal fluid or through percutaneous transhepatic cholangiography and drainage (PTCD) to drain both the intrahepatic biliary ducts and the jejunal loop close to the anastomoses. The expert panel is available to provide advice on indication and feasibility of PTCD. Surgical intervention through relaparotomy for hepaticojejunostomy leak any time 24 hours after index surgery is only indicated when the patient remains critically ill with radiologic optimal drainage[33].

**Addendum: postpancreatectomy bleeding**

When the clinical suspicion of postpancreatectomy bleeding rises (i.e. fresh blood in abdominal catheter or nasogastric tube, drop haemoglobin or otherwise suspicion of abdominal bleeding), a diagnostic CT-angiography should be performed. If the source of the bleeding (i.e. contrast extravasation or pseudo-aneurysm) is visualized on CT, selective angiography should be performed with possible stent-graft placement or coiling. If no source of bleeding is seen on CT, but the clinical suspicion of haemorrhage remains, a subsequent selective diagnostic angiography of the coeliac axis and superior mesenteric artery should be considered. Surgical intervention is only indicated in the early postoperative phase (<24h after index resection; left to the surgeons discretion) or if the patient remains haemodynamically unstable with no (further) angiographic options to control the bleeding[34].

**Addendum: organ failure**

In case of new-onset (multi-)organ failure patients should be admitted to the intensive care unit (ICU). Carefully monitoring these patients and supporting the failing organs is then indicated. Patients who deteriorate with clinical signs of infection require cultures of blood, urine, sputum, ascites and frequent diagnostic imaging (e.g. chest X-rays amongst others). Once an infectious focus is determined, targeted antibiotics should be given or, in case of no positive culture and persisted deterioration, broad spectrum antibiotics with optimal penetration must be started[25].

**REFERENCES**

1. Smits FJ, Molenaar IQ, Besselink MG, Borel Rinkes IHM, van Eijck CHJ, Busch OR, et al. Early recognition of clinically relevant postoperative pancreatic fistula: a systematic review. HPB (Oxford). Elsevier; 2019;0.

2. Bassi C, Marchegiani G, Dervenis C, Sarr M. The 2016 update of the International Study Group ( ISGPS ) definition and grading of postoperative pancreatic fistula : 11 Years After. Elsevier Inc.; 2016;1–8.

3. Biffl WL, Moore EE, Moore FA, Peterson VM. Interleukin-6 in the injured patient. Marker of injury or mediator of inflammation? Ann Surg. 1996;224:647–64.

4. Noji T, Nakamura T, Ambo Y, Suzuki O, Nakamura F, Kishida A, et al. Clinically relevant pancreas-related infectious complication after pancreaticoenteral anastomosis could be predicted by the parameters obtained on postoperative day 3. Pancreas. 2012;41:916–21.

5. Moskovic DJ, Hodges SE, Wu M-F, Brunicardi FC, Hilsenbeck SG, Fisher WE. Drain data to predict clinically relevant pancreatic fistula. HPB [Internet]. 2010 [cited 2017 Apr 14];12:472–81. Available from: http://doi.wiley.com/10.1111/j.1477-2574.2010.00212.x

6. Uemura K, Murakami Y, Sudo T, Hashimoto Y, Kondo N, Nakagawa N, et al. Indicators for proper management of surgical drains following pancreaticoduodenectomy. J Surg Oncol. 2014;109:702–7.

7. Giardino A, Spolverato G, Regi P, Frigerio I, Scopelliti F, Girelli R, et al. C-Reactive Protein and Procalcitonin as Predictors of Postoperative Inflammatory Complications After Pancreatic Surgery. J Gastrointest Surg. 2016;20:1482–92.

8. Kosaka H, Kuroda N, Suzumura K, Asano Y, Okada T, Fujimoto J. Multivariate logistic regression analysis for prediction of clinically relevant pancreatic fistula in the early phase after pancreaticoduodenectomy. J Hepatobiliary Pancreat Sci [Internet]. 2014 [cited 2017 Apr 14];21:128–33. Available from: http://www.ncbi.nlm.nih.gov/pubmed/23804410

9. Fujiwara Y, Shiba H, Shirai Y, Iwase R, Haruki K, Furukawa K, et al. Perioperative serum albumin correlates with postoperative pancreatic fistula after pancreaticoduodenectomy. Anticancer Res. 2015;35:499–503.

10. Furukawa K, Gocho T, Shirai Y, Iwase R, Haruki K, Fujiwara Y, et al. The Decline of Amylase Level of Pancreatic Juice After Pancreaticoduodenectomy Predicts Postoperative Pancreatic Fistula. Pancreas. 2016;45:1474–7.

11. Palani Velu LK, Chandrabalan V V., Jabbar S, McMillan DC, McKay CJ, Carter CR, et al. Serum amylase on the night of surgery predicts clinically significant pancreatic fistula after pancreaticoduodenectomy. HPB [Internet]. 2014;16:610–9. Available from: http://www.ncbi.nlm.nih.gov/pubmed/24246024

12. Ansorge C, Nordin JZ, Lundell L, Strömmer L, Rangelova E, Blomberg J, et al. Diagnostic value of abdominal drainage in individual risk assessment of pancreatic fistula following pancreaticoduodenectomy. Br J Surg. 2014;101:100–8.

13. Solaini - inflammatory markers in early detection of postoperative inflammatory complications - Int J Surg 2015.

14. Solaini L, Atmaja BT, Watt J, Arumugam P, Hutchins RR, Abraham AT, et al. Limited utility of inflammatory markers in the early detection of postoperative inflammatory complications after pancreatic resection: Cohort study and meta-analyses. Int J Surg [Internet]. Elsevier Ltd; 2015 [cited 2017 Apr 14];17:41–7. Available from: http://dx.doi.org/10.1016/j.ijsu.2015.03.009

15. Bone RC, Balk RA, Cerra FB, Dellinger RP, Fein AM, Knaus WA, et al. Definitions for Sepsis and Organ Failure and Guidelines for the Use of Innovative Therapies in Sepsis. Chest. American College of Chest Physicians; 1992;101:1644–55.

16. Bruno O, Brancatelli G, Sauvanet A, Vullierme MP, Barrau V, Vilgrain V. Utility of CT in the diagnosis of pancreatic fistula after pancreaticoduodenectomy in patients with soft pancreas. Am J Roentgenol. 2009;193:W175-80.

17. Hashimoto M, Koga M, Ishiyama K, Watarai J, Shibata S, Sato T, et al. CT features of pancreatic fistula after pancreaticoduodenectomy. AJR Am J Roentgenol. 2007;188:W323-7.

18. Smits FJ, van Santvoort HC, Besselink MG, Batenburg MCT, Slooff RAE, Boerma D, et al. Management of Severe Pancreatic Fistula After Pancreatoduodenectomy. JAMA Surg [Internet]. 2017 [cited 2017 Mar 7]; Available from: http://www.ncbi.nlm.nih.gov/pubmed/28241220

19. Malleo G, Pulvirenti A, Marchegiani G, Butturini G, Salvia R, Bassi C. Diagnosis and management of postoperative pancreatic fistula. Langenbecks Arch Surg. 2014;399:801–10.

20. Tol JAMG, Busch ORC, van Delden OM, van Lienden KP, van Gulik TM, Gouma DJ. Shifting role of operative and nonoperative interventions in managing complications after pancreatoduodenectomy: what is the preferred intervention? Surgery. 2014;156:622–31.

21. King JC, Hogg M, Zeh HJ. Major Disruptions of Pancreaticojejunostomy. Gastrointest Surg. New York, NY: Springer New York; 2015. p. 281–92.

22. Sohn TA, Yeo CJ, Cameron JL, Geschwind JF, Mitchell SE, Venbrux AC, et al. Pancreaticoduodenectomy: role of interventional radiologists in managing patients and complications. J Gastrointest Surg. 2003;7:209–19.

23. Sanjay P, Kellner M, Tait IS. The role of interventional radiology in the management of surgical complications after pancreatoduodenectomy. HPB (Oxford). 2012;14:812–7.

24. van Grinsven J, van Brunschot S, van Santvoort HC, Schepers NJ, Doeve B, Bakker OJ, et al. The Value of a 24/7 Online Nationwide Multidisciplinary Expert Panel for Acute Necrotizing Pancreatitis. Gastroenterology. 2017;152:685–688.e6.

25. Behrman SW, Bahr MH, Dickson P V, Zarzaur BL. The Microbiology of Secondary and Postoperative Pancreatic Infections. Arch Surg. 2011;146:613.

26. McMillan MT, Malleo G, Bassi C, Allegrini V, Casetti L, Drebin JA, et al. Multicenter, prospective trial of selective drain management for pancreatoduodenectomy using risk stratification. Ann Surg. 2017;265:1209–18.

27. Tsujie M, Nakamori S, Miyamoto AA, Yasui M, Ikenaga M, Hirao M, et al. Risk Factors of PAncreatic Fistula after Pancreaticoduodenectomy - Patients with Low Drain Amylase Level on Postoperative Day 1 are Safe from Developing Pancreatic Fistula. Hepatogastroenterology. 2012;59:2657–60.

28. Dugalic VD, Knezevic DM, Obradovic VN, Gojnic-Dugalic MG, Matic S V, Pavlovic-Markovic AR, et al. Drain amylase value as an early predictor of pancreatic fistula after cephalic duodenopancreatectomy. World J Gastroenterol. 2014;20:8691–9.

29. Kobayashi S, Gotohda N, Kato Y, Takahashi S, Konishi M, Kinoshita T. Infection control for prevention of pancreatic fistula after pancreaticoduodenectomy. Hepatogastroenterology. 2013;60:876–82.

30. El Nakeeb A, Salah T, Sultan A, El Hemaly M, Askr W, Ezzat H, et al. Pancreatic Anastomotic Leakage after Pancreaticoduodenectomy. Risk factors, Clinical predictors, and Management (Single Center Experience). World J Gastroenterol. 2013;37.

31. Shinchi H, WADA K, TRAVERSO LW. The Usefulness of Drain Data to Identify a Clinically Relevant Pancreatic Anastomotic Leak After Pancreaticoduodenectomy? J Gastrointest Surg [Internet]. 2006 [cited 2017 Apr 14];10:490–8. Available from: http://linkinghub.elsevier.com/retrieve/pii/S1091255X0500661X

32. Facy O, Chalumeau C, Poussier M, Binquet C, Rat P, Ortega-Deballon P. Diagnosis of postoperative pancreatic fistula. Br J Surg [Internet]. 2012 [cited 2017 Apr 26];99:1072–5. Available from: http://www.ncbi.nlm.nih.gov/pubmed/22539219

33. Koch M, Garden OJ, Padbury R, Rahbari NN, Adam R, Capussotti L, et al. Bile leakage after hepatobiliary and pancreatic surgery: a definition and grading of severity by the International Study Group of Liver Surgery. Surgery [Internet]. 2011 [cited 2015 Nov 12];149:680–8. Available from: http://www.ncbi.nlm.nih.gov/pubmed/21316725

34. Malleo G, Vollmer CM. Postpancreatectomy Complications and Management. Surg Clin North Am [Internet]. Elsevier Inc; 2016;96:1313–36. Available from: http://dx.doi.org/10.1016/j.suc.2016.07.013
